# Enhanced dynamic coupling in a nuclear receptor underlies ligand activity

**DOI:** 10.1016/j.jbc.2024.108081

**Published:** 2024-12-14

**Authors:** Tracy Yu, Priscilla Villalona, Sabab Hasan Khan, Noriko Mikeasky, Emily Meinert, Jill Magafas, Thilini Pulahinge, Ameen Bader, C. Denise Okafor

**Affiliations:** 1 Department of Biochemistry and Molecular Biology, Pennsylvania State University, University Park, Pennsylvania, USA; 2 Department of Chemistry, Pennsylvania State University, University Park, Pennsylvania, USA

**Keywords:** farnesoid X receptor, nuclear receptors, molecular dynamics simulations, allosteric signaling, coregulators, transcriptonal regulation, bile acids, agonists

## Abstract

Bile acids are signaling molecules with critical roles in cholesterol and lipid metabolism, achieved by regulating the transcriptional activity of the farnesoid X receptor (FXR, NR1H4), otherwise known as the bile acid receptor. Modifications to the C6 position of the steroidal core yield bile acid derivatives with 100× improved potency over endogenous bile acids. Prevailing hypotheses suggested increased binding affinity for FXR as the driver for this activity enhancement. Our experimental results contradict this suggestion, motivating us to investigate the underlying mechanisms of enhanced ligand activity. We combined functional assays with over 200 μs of simulations, revealing an unexpected role for helix 5 in the allosteric signaling of obeticholic acid. We uncovered dynamic coupling between adjacent helices 5 and 7, which is uniquely enhanced by the bile acid modification. Ultimately, the enhanced potency of the bile acid analog can be traced to its effect on FXR dynamics. In addition to identifying a previously unknown mechanistic role for helix 5 to helix 7 coupling in FXR, these results emphasize the inextricable linkage between the activity of nuclear receptor ligands and their effects on receptor dynamics.

The farnesoid X receptor (FXR, NR1H4), a member of the nuclear receptor superfamily, is known for its crucial role in the regulation of bile acid, lipid, and glucose metabolism (1, 2). As a ligand-regulated transcription factor, FXR is responsive to bile acids, the amphipathic end products of cholesterol catabolism (2, 3, 4). FXR is highly expressed in the liver and intestine (5). The human bile acid pool includes primary bile acids, cholic acid, and chenodeoxycholic acid (CDCA) (6). The major secondary bile acids, deoxycholic acid and lithocholic acid (LCA), are formed by 7-dehydroxylation of cholic acid and CDCA, respectively, by intestinal bacteria (7). CDCA is the most potent endogenous activator of FXR, with an EC50 value of approximately 10 μM (8). Other bile acids display weaker potency, ranging from antagonism to partial agonism (9). For example, LCA is a weak agonist (10), displaying antagonism in some *in vitro* assays (11) (EC50 = 50 μM) (3). Bile acids can also be conjugated with glycine and taurine, or other amino acids, which are found to bind and activate FXR (3).

Due to its integral role in metabolism, FXR is a drug target for several diseases, including nonalcoholic steatohepatitis (12), also known as metabolic dysfunction–associated steatohepatitis (13), primary biliary cholangitis (14), type II diabetes (2) and atherosclerosis (15). Several FXR agonists are currently undergoing clinical trials for the treatment of nonalcoholic steatohepatitis, and cholestatic liver diseases, including tropifexor (LJN452) (16, 17), cilofexor (GS9674 or Px201) (16, 17, 18, 19), nidufexor (20), and MET409 (21). While these FXR agonists have shown promise in clinical trials, they are associated with class-related side effects. These side effects include pruritus, increased cholesterol plasma levels, reduced high-density lipoprotein, and variable effects on liver steatosis and fibrosis (22, 23). Understanding the specific mechanisms of FXR activation by ligands is crucial for developing selective FXR modulators that maximize therapeutic efficacy while minimizing undesirable side effects.

Despite extensive studies on how ligands regulate the transcriptional activity of FXR (9), the mechanisms driving potent activation in FXR remain poorly understood. Like other nuclear receptors, FXR undergoes conformational changes in response to ligand binding that regulate and modulate multiple events, including recruitment of coregulators and heterodimer formation with retinoid X receptor alpha (RXRα) (24). Nuclear receptor coregulators (25), for example, peroxisome proliferator–activated receptor γ coactivator 1α (PGC-1α), steroid receptor coactivators (SRCs), are critical components of transcriptional complexes, contributing to either upregulation or downregulation of transcription (26). Coregulators are recruited to the activation function 2 (AF-2) surface located on the ligand-binding domain (LBD) (27). In FXR, ligands modulate coregulator recruitment in dose-specific manners (9, 24, 28, 29, 30, 31, 32, 33, 34). Ligand binding also influences dimerization between FXR and RXR, with different effects observed depending on the ligand scaffold (29, 35, 36). As FXR primarily binds DNA as a heterodimer, transcription is dependent on this dimerization (24, 37). Further, both coregulator binding and dimerization with RXR were demonstrated to be tightly linked with ligand binding in FXR (24). Thus, it is a reasonable hypothesis that the potency of FXR ligands is related to how they modulate coregulator recruitment and/or heterodimer formation.

Building on our understanding of FXR ligands in modulating coregulator recruitment and dimerization, an important question arises: does efficacy in coregulator recruitment directly translate to efficacy in promoting FXR-RXR dimerization? Do potent agonists enhance both of these activities compared to weaker ligands? An exhaustive study (35) on FXR ligands with diverse scaffolds demonstrated that neither of these events represents an isolated mechanism for potent modulation of FXR. Some agonists do both well, while others promote either coregulator recruitment or dimer formation. Thus, an ensemble of effects appeared to govern FXR modulation, confirming a complex interplay between the two mechanisms, modulated by ligand binding. This ability of ligands to influence coregulator association and dimerization in unique ways hints at the complexity of ligand-induced dynamics in nuclear receptors. Indeed, depending on their structure, ligands produce distinct effects on the conformational dynamics of receptors, which subsequently affects both protein structure and function (35, 38), also reported in FXR (36, 39, 40). Therefore, we are not likely to completely understand the origin of ligand potency without deciphering the impact of ligand structures on receptor dynamics.

Our goal is to understand the specific aspects of ligand structure that allow them to be potent agonists, as well as the mechanism underlying enhanced activity. We propose that an effective way to tackle this question is to focus on learning how a simple modification to a ligand enhances its activity, including coregulator and dimer interactions. To achieve this goal, we must identify ligands with appreciable differences in potency accompanied by minor changes in molecular structure. Similar approaches have been applied to identify structural or biochemical mechanisms in a series of ligands targeting glucocorticoid receptor (41), estrogen receptor (42), peroxisome proliferator-activated receptor gamma (43), and liver receptor homolog-1 (44), yielding remarkable insight into how ligand modifications influence receptor activity. Extensive structure-activity relationship studies of bile acids have identified modifications that enhance their potency in FXR (45, 46, 47). These ligands provide a starting point for learning how small structural changes specifically increase their activity.

Our particular focus is on obeticholic acid (OCA, marketed as OCALIVA), the 6-ethyl derivative of CDCA (Fig. 1*A*), which along with other 6-alpha-alkylated CDCA homologs, was shown to increase both potency and efficacy compared to CDCA (48, 49, 50). Following its discovery in 2002, OCA (EC50 = 99 nM) (50) exhibited promising therapeutic potential, particularly in addressing cholestatic liver diseases (51). Despite its clinical benefits, certain adverse effects were reported with OCA use, including pruritus, gallstones, and acute cholecystitis (51, 52). While the use of OCA has now been discontinued for several ailments (fda.gov.), it remains a valuable model for identifying the molecular mechanism by which the 6-ethyl group enhances potency in FXR.

**Figure 1:**
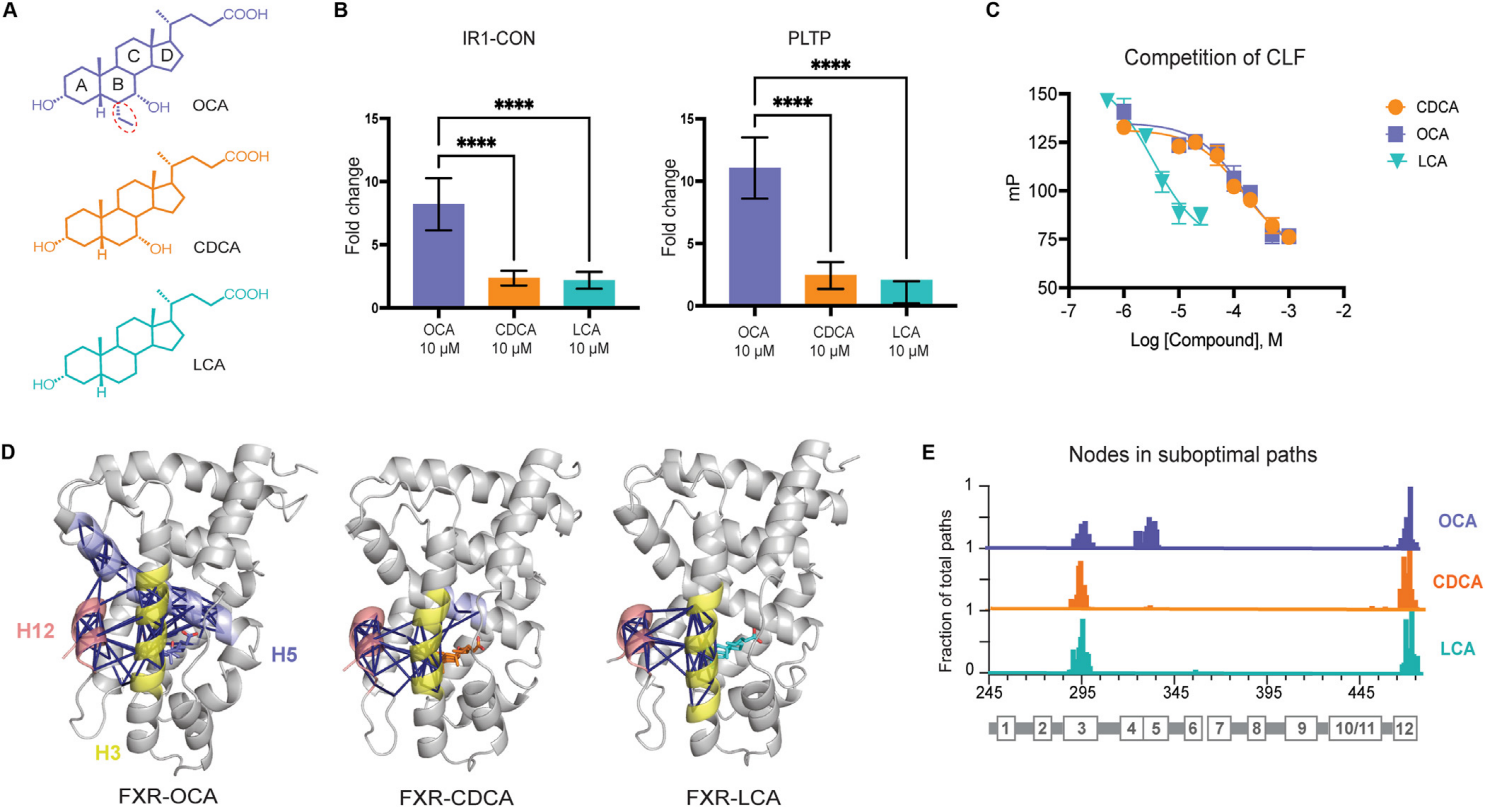
**Comparison of the structure, activity, and dynamics of OCA, CDCA, and LCA.***A*, structures of OCA (*top*, in *purple*), CDCA (*middle*, in *orange*), and LCA (*bottom*, in *cyan*). *B*, luciferase activity assays of FXR using both IR-1-CON-*luc* and PLTP-*luc* with 10 μM of OCA, CDCA, and LCA. OCA has the highest efficacy in both assays. *C*, binding affinities of the bile acids were determined using fluorescence polarization assays. The Ki values are 150.6 μM for OCA, 140.2 μM for CDCA, and 3.283 μM for LCA. *D*, structures of FXR-LBD in complex with the three bile acids, showing suboptimal paths between ligand and H12 residue E471. Helices involved in communication include H3 (in *yellow*), H5 (in *light blue*), and H12 (in *salmon*). OCA displays extensive utilization of H5 residues compared to other bile acids. *E*, histograms depicting the residues involved in suboptimal paths between the ligand and H12. The *y*-axis indicates a normalized percentage utilization of each residue relative to the total combined number of paths between the ligand and H12. While CDCA and LCA show high usage of H3 residues, this frequency is reduced in OCA along with the incorporation of H5 residues. CDCA, chenodeoxycholic acid; FXR, farnesoid X receptor; LBD, ligand-binding domain; LCA, lithocholic acid; OCA, obeticholic acid.

To determine how OCA achieves higher activity than natural bile acids CDCA and LCA, we first focus on studying how these ligands impact FXR dynamics. Molecular dynamics (MD) simulations immediately reveal that helix 5 (H5) of the FXR LBD is selectively modulated by OCA. Mutagenesis of H5 reduces OCA efficacy, confirming a special role for the helix in OCA activity. Experimental measurements of FXR coregulator and FXR–RXR interactions do not readily reveal the origin of the H5 effect. We show that OCA dynamically rewires allosteric communication in the LBD, incorporating heavy utilization of H5. This effect can be traced to helix 7 (H7), which directly contacts the 6-ethyl group of OCA. We provide both experimental and *in silico* evidence of dynamic coupling between the two helices, which is enhanced by OCA. Other bile acids do not enhance this coupling to the same extent, and potent nonbile acid ligands do not support the H5 mechanism. These findings emphasize that FXR activity can be modulated by diverse mechanisms and underscore the importance of studying receptor-ligand dynamics, as these can explain the origin of ligand potency.

## Results

### MD simulations predict a role for helix 5 in OCA activity

While they share the same steroidal scaffold (Fig. 1*A*), bile acids exert wildly different effects on FXR. To quantify baseline activation levels at 10 μM, we measured FXR transcription by OCA, CDCA, and LCA using two luciferase reporter constructs: IR-1-CON-*luc* and PLTP-*luc* (see Methods) (Fig. 1*B*). In this work, we quantify ligand activity by measuring efficacy of FXR transcriptional activation rather than potency (*i.e.* EC_50_), both of which are reported in published studies to be enhanced in OCA over CDCA. For both reporter constructs, we observed significantly higher activity in OCA than in CDCA and LCA and no marked difference between the latter two at 10 μM. Raising [LCA] and [CDCA] from 10 μM to 100 μM (Fig. S1*A*) increases their activity but still falls short of 10 μM OCA levels, emphasizing the enhanced efficacy of OCA over CDCA and LCA.

Structural studies have implied that the ethyl group of OCA enhances its binding affinity, resulting in its increased activity (32, 50). This group protrudes into the hydrophobic cavity formed by H7, interacting with side chains of I366, F370, and Y373. To test the hypothesis that the increased activity of OCA results from enhanced affinity due to hydrophobic interactions in the pocket, we performed fluorescence polarization competition experiments. We assessed ligand binding by outcompeting a fluorescent bile acid, cholyl-lys-fluorescein (CLF) (Fig. 1*C*). Our findings indicated that despite its weaker activity, LCA binds FXR more tightly than CDCA and OCA. Further, no significant difference is observed in the binding affinity of CDCA and OCA, suggesting that the efficacy of OCA does not result from increased binding affinity. This result further supports previous findings showing that binding affinity does not consistently predict the activity of nuclear receptor ligands (43).

To learn how OCA achieves its efficacy in FXR activation, we performed MD simulations of FXR in complex with the three bile acids. We performed a suboptimal paths analysis with the goal of examining how different bile acids modulate communication and signaling between the ligand binding pocket and AF-2, approximated by E471 (H12) (Fig. 1*D*). Notably, the OCA complex generated 30-fold more communication pathways than those of CDCA and LCA within a cut-off of 100 (see Methods). The broadest signaling is observed in OCA, with communication extensively traversing both H3 and H5, unlike LCA and CDCA, which primarily use H3. We quantified the node residues appearing in the shortest 1000 paths, confirming that over 50% of paths in CDCA and LCA complexes traverse H3 (Fig. 1*E*). In contrast, less than 50% in FXR-OCA utilize H3, with the majority of paths instead incorporating H5 residues (residues 330–340). Finally, we quantified the length of these 1000 suboptimal paths, a metric assumed to be inversely proportional to the strength of communication between two sites (Fig. S1*B*). The shortest path lengths are observed in OCA, followed by CDCA and LCA. These findings indicate that ligands modulate dynamics and predicted communication in FXR, suggesting a correlation between ligand efficacy and the strength of communication. The results also suggest a unique role for H5 in mediating OCA activity.

### Helix 5 mutations selectively impact OCA efficacy

**Figure 2:**
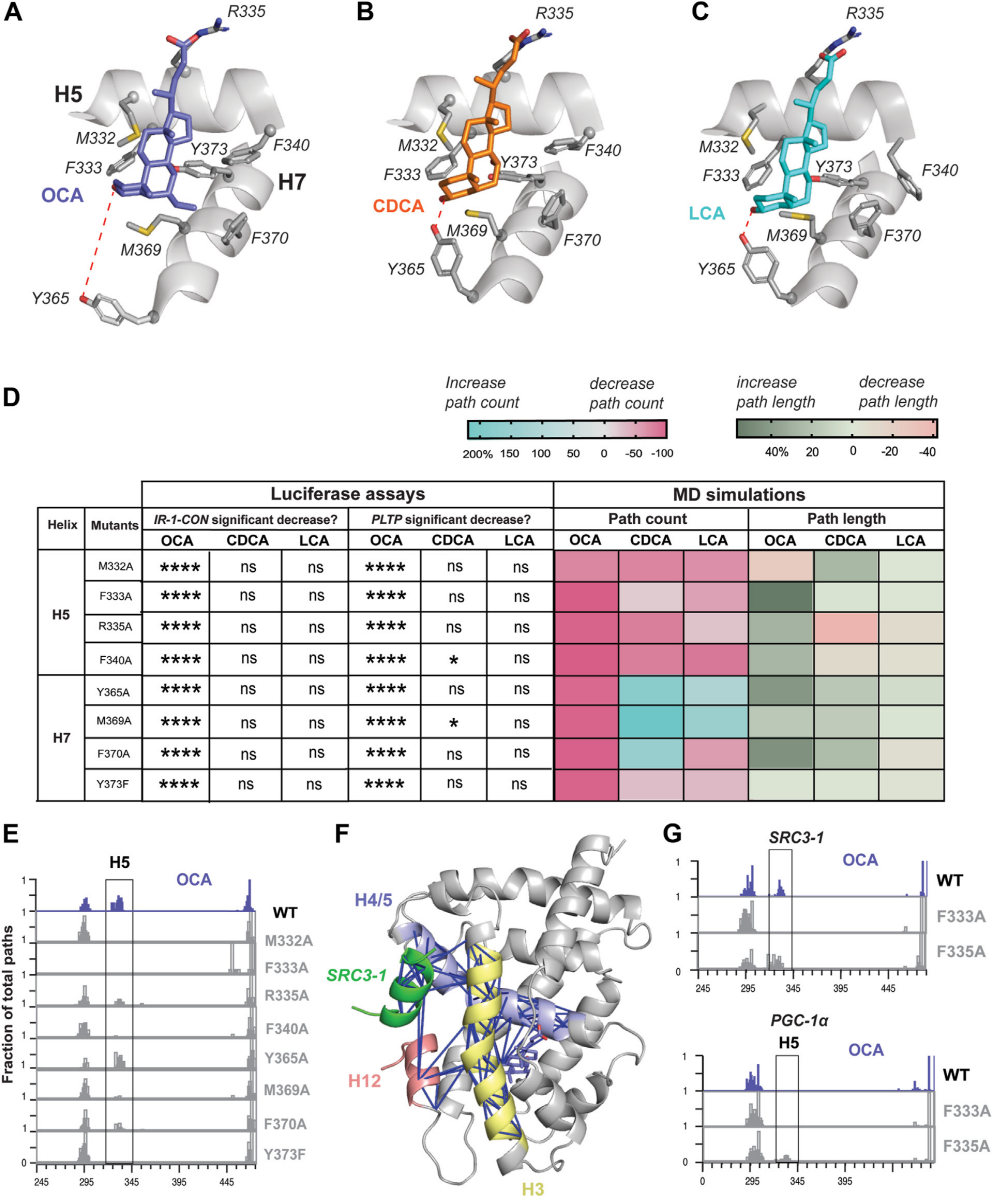
**The role of H5 residues in FXR activity.***A–C*, bile acids in the FXR ligand binding pocket depicting interactions with the H5 and H7 residues. The average distances between Y365 and each bile acid are as follows: Y365-OCA (6.78 Å), Y365-CDCA (2.99 Å), and Y365-LCA (3.15 Å). *D*, summary table showing the effect of H5 and H7 mutations, assessed through luciferase assays and MD simulations. Luciferase assays demonstrate the impact of H5 and H7 mutations on FXR transactivation of IR-1-CON-*luc* and PLTP-*luc*. MD simulations assess the impact of these mutations on the communication between the ligand and the AF-2, quantified by the number of paths (path count) and length of paths, in comparison to WT. Path counts identify the percent increase (*cyan*) or decrease (*magenta*) in the number of paths relative to the WT complex. Path lengths quantify the percent increase (*green*) or decrease (*pink*) in path lengths relative to the WT complex. *E*, histograms depicting the residues involved in suboptimal paths between ligand and H12 of WT, H5, and H7 mutants bound to OCA. *F*, model of FXR-LBD bound to OCA and SRC3-1. *G*, histograms depicting the residues involved in suboptimal paths between ligand and coregulator peptides. Comparison is made between WT and two H5 mutants, F333A and F340A, in complex with OCA and coregulators, PGC-1α and SRC3-1. AF-2, activation function 2; CDCA, chenodeoxycholic acid; FXR, farnesoid X receptor; LBD, ligand-binding domain; MD, molecular dynamics; OCA, obeticholic acid; PGC-1α, peroxisome proliferator–activated receptor γ coactivator 1α; SRC, steroid receptor coactivator.

To determine whether H5 plays a role in OCA activity, we created a set of H5 FXR mutants, including M332A, F333A, R335A, and F340A. We also designed H7 mutants M369A and F370A, with the goal of disrupting interactions between the ethyl tail of OCA and H7 (Fig. 2*A*). Y365A and Y373F mutants were created to disrupt hydrogen bonding between H7 and bile acids (Fig. 2, *A–C*). Our simulations identified Y365 as a residue of interest, based on its consistent hydrogen bonding with CDCA (97%) and LCA (93%), which was lacking in OCA (43%). We tested the activity of all mutants using the IR-1-CON-*luc* reporter, observing that all H5 mutations reduced OCA-induced transcriptional levels, while not impacting CDCA or LCA activity (Figs. 2*D* and S2*A*). We repeated this assay with PLTP-*luc* where we observed nearly identical trends (Figs. 2*D* and S2*A*). The only exception was a small decrease in CDCA efficacy in the F340A mutant. The H7 mutations introduced a similar decrease in OCA efficacy in both IR-1-CON-*luc* and PLTP-*luc* reporters. CDCA and LCA activity remained largely unaffected by H7 mutations, but a minor effect was observed in CDCA activity in the M369A mutant. We tested higher concentrations of CDCA and LCA as well (Fig. S2*B*). At 100 μM, H5 and H7 mutations significantly reduced CDCA activity for both luciferase reporters, except for the F340A mutant, which did not affect CDCA activity in the IR-1-CON-*luc* assay. LCA activity is unaffected with IR-1-CON-*luc* but shows decreased activity in the PLTP-*luc* assay. These findings confirm that H5 and H7 are specifically connected to the efficacy of OCA.

To reveal the dynamic effects of the H5 and H7 mutations, we generated *in silico* mutants, each complexed with OCA, CDCA, and LCA. We obtained MD trajectories for each complex and analyzed suboptimal paths between the ligand and H12. We quantified the number of paths within the 100 Å cut-off (see Methods), comparing these to the equivalent WT FXR complex. In all OCA complexes, H5 and H7 mutants significantly decrease the path counts (Fig. 2*D*). These effects were attenuated in CDCA and LCA complexes. We also measured the lengths of the shortest 1000 paths for each mutant complex and performed a similar comparison with WT FXR. Except for M332A, which showed a reduction in path lengths, H5 and H7 mutations increased path lengths in OCA complexes, consistent with weakened signaling (Fig. 2*D*). Path lengths also increase in LCA and CDCA mutant complexes, but to a lesser extent. While these data do not directly correlate with experiments, they consistently suggest that compared to the other ligands, the dynamic behavior of OCA-bound FXR is most impacted by H5 and H7 mutations.

We hypothesized that H5 and H7 mutations might also alter the residue preference of suboptimal paths, particularly reducing the involvement of H5 in communication. To test this hypothesis, we performed a by-residue decomposition of the shortest 1000 paths for each complex (Figs. 2*E* and S3*A*). We observed that the involvement of H5 in WT FXR-OCA is reduced in all H5 mutants and two of the H7 mutants (Fig. 2*E*). In contrast, while CDCA and LCA do not engage H5 residues in the WT receptor, several H7 and H5 mutants unexpectedly gain H5 involvement (Fig. S3*A*). This might be indicative of coupling that naturally exists between the two helices when any bile acid is bound. We also used the shortest distance analysis (see Methods) to characterize the relationship between ligands and surrounding residues (Fig. S3*B*). This analysis uses covariances calculated from MD trajectories to approximate the strength of communication between pairs of residues. The shortest distance analysis reveals stronger coupling (*i.e.*, smaller shortest distance values) with H5 residues, which is mitigated by H5 mutations. This effect is not replicated in LCA or CDCA complexes. Further, other FXR binding pocket residues (on H7, H3, H10, and H12) do not display similarly strong coupling with H5, or any effects upon mutation. This analysis underscores the unique role of H5 in signaling involving OCA.

To further assess the role of H5 in FXR dynamics, we added peptide fragments of nuclear receptor coregulators SRC-3 (SRC3-1), SRC-2, and PGC-1α to our models of WT FXR (Fig. 2*F*). We also generated complexes with H5 mutants F333A and F340A, which we selected because they showed the largest change in shortest distance values (Fig. S3*B*). We generated 27 unique complexes (3 peptides × 3 ligands ×3 FXR variants) and obtained triplicate MD trajectories for all. We identified suboptimal paths between the ligand and peptide, hypothesizing that H5 mutations would affect the direction and strength of the paths. Of the three peptides tested, SRC3-1 was the only complex where OCA signaled to the peptide through H5 (Fig. 2*G*). While H5 involvement is attenuated in the F333A mutant complex, no change is observed in F340A. When the suboptimal paths analysis is repeated using H12 as the “sink” (see Methods), no differences in the results are observed. These findings suggest that the identity of coregulator peptides influences the dynamics and signaling patterns in OCA-bound FXR. Regardless, we confirm that mutating H5 disrupts the involvement of H5 residues in FXR communication.

### Distinct roles for helix 5 and helix 7 in FXR activity

Simulations and transactivation experiments establish an unexpected role for H5 in OCA activation. To understand the specific role of H5 in FXR activity, we sought to determine how mutating H5 affects the interaction between FXR and coregulators, as well as between FXR and RXR. Using a mammalian two-hybrid system (M2H), we generated chimeric constructs of FXR LBD (VP16-FXR-LBD) and coactivator PGC-1α (Gal4DBD-PGC-1α) in pACT and pBIND vectors, respectively (see Methods). We also generated a chimeric construct with the SRC1-2 coactivator fragment (Gal4DBD-SRC1-2), which has been shown to interact with FXR (35). Along with H5 mutations F333A and F340A, we generated a H7 mutant F370A of the LBD construct. F370 was selected from H7, based on having the highest proximity to the OCA ethyl tail.

**Figure 3:**
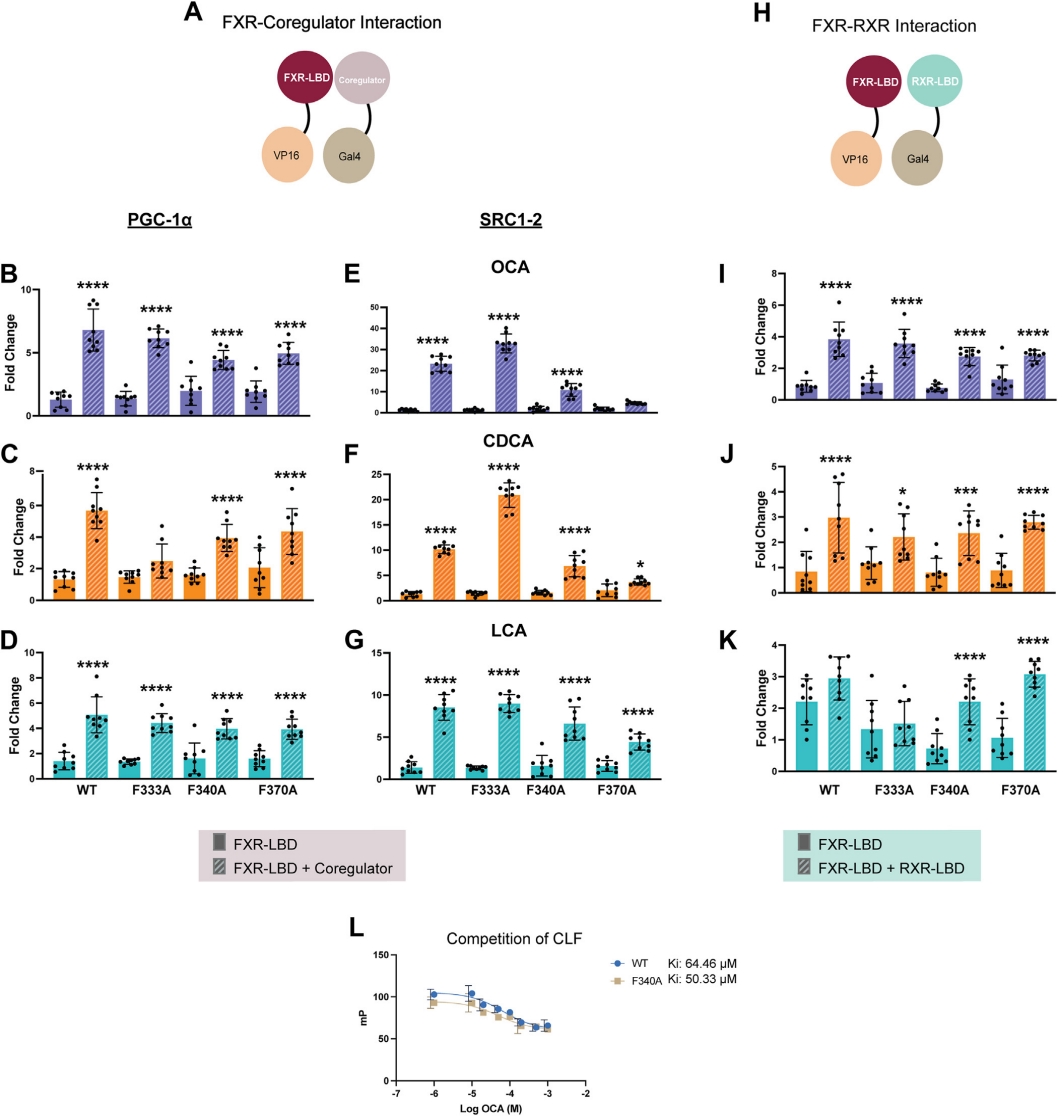
**Effect of H5 and H7 mutations on FXR-coregulator and FXR–RXR interactions.***A*, schematic representations of the hybrid constructs used in the M2H assay to quantify the interaction between FXR and coregulator. FXR LBD (*maroon*) is fused to VP16, while PGC-1α and SRC1-2 (*pink*) is fused to the Gal4-DBD. *B–D*, luciferase assays testing the interaction between WT and mutant (F333A, F340A, and F370A) FXR constructs with PGC-1α coregulator. *E–G*, luciferase assays testing the interaction between WT and mutant (F333A, F340A, and F370A) FXR constructs with SRC1-2 coactivator fragment. Ligand concentrations of OCA, CDCA, and LCA are 10 μM. H5 mutation (F333A) disrupted the interaction between FXR and PGC-1α in CDCA-treated cells. H7 mutation (F370A) disrupted the interaction between FXR and the SRC1-2 coactivator fragment in OCA- and CDCA-treated cells with RXR transfected. *H*, schematic representation of the hybrid constructs used in the M2H assay to quantify the interaction between FXR and RXR. RXR (*teal*) is fused to the Gal4-DBD. *I–K*, luciferase assays testing the interaction between WT and mutant (F333A, F340A, and F370A) FXR constructs with RXR. With both H5 and H7 mutations the interaction between FXR and RXR was not disrupted. *L*, fluorescence polarization-based competitive binding assay for FXR-LBD-MBP (*blue*) and F340A FXR-LBD-MBP (*tan*) using OCA to outcompete CLF. The mutation did not have an impact on the binding affinity of OCA for the WT, with a Ki of 64.46 μM for the WT and 50.33 μM for the mutant. CDCA, chenodeoxycholic acid; CLF, cholyl-lys-fluorescein; DBD, DNA-binding domain; FXR, farnesoid X receptor; LBD, ligand-binding domain; LCA, lithocholic acid; M2H, mammalian two-hybrid system; MBP, maltose-binding protein; MD, molecular dynamics; OCA, obeticholic acid; PGC-1α, peroxisome proliferator–activated receptor γ coactivator 1α; RXR, retinoid X receptor; SRC, steroid receptor coactivator.

Using the M2H system, we measured the ligand-induced interaction between FXR and PGC-1α, comparing WT and mutant FXR constructs. Without cotransfection of RXR, ligands did not appear to induce an interaction between FXR and PGC-1α in our assay (Fig. S4*A*). With RXR cotransfection, we observe the FXR–PGC-1α interaction, noting that H5 mutations do not disrupt the interaction for OCA- and LCA-treated cells (Fig. 3, *B–D*). However, with CDCA, the F333A H5 mutation reduces the coregulator interaction (Fig. 3*C*). We repeated the experiment using the SRC1-2 coactivator fragment, which weakly interacts with FXR in the absence of RXR (Fig. S4*B*). Interestingly, only OCA-treated cells revealed the interaction between FXR and SRC1-2, and both H5 mutants significantly decreased this interaction in the M2H assay (Fig. S4*B*). When RXR was cotransfected, all ligands induced interactions between FXR and SRC1-2 (Fig. 3, *E–G*). For OCA and CDCA, we observe a loss of the FXR–coregulator interaction in the F340A mutant (Fig. 3, *E* and *F*). We tested the H7 mutant in the M2H assay with both coregulators and did not observe a disruption of the FXR interaction with PGC-1α (Fig. 3, *B–D*). However, the H7 mutant disrupted the interaction between SRC1-2 and FXR for OCA- and CDCA-treated cells when RXR was cotransfected (Fig. 3, *E* and *F*). The interaction in OCA-treated cells was also disrupted without RXR cotransfection (Fig. S4*B*). To further assess the effect of H5 mutations on coregulator recruitment to FXR, we used an alternate approach where we transfected PGC-1α and directly measured its impact on FXR transactivation with IR-1-CON-*luc* and PLTP-*luc* (Fig. S5*A*). Consistent with the results of the M2H assay, H5 mutations did not disrupt the interaction between FXR and PGC-1α (Fig. S5*A*). For the transfection of full SRC1 and SRC3, we did not observe any increase in transcriptional activity, even with WT FXR (Fig. S5, *B* and *C*).

To test other mechanisms by which H5 mutations might disrupt FXR activity, we used the M2H assay to assess FXR-RXR heterodimerization. We generated a Gal4DBD-RXR-LBD chimera, allowing us to measure the interaction between RXR and FXR LBD. Similar to the FXR–coregulator interaction, we do not observe an effect of H5 mutations on the FXR–RXR interaction (Fig. 3, *I–K*). We also tested the F370A (H7 mutant) in this assay and did not observe a disruption in the FXR–RXR interaction. This effect is observed across all ligands, suggesting that H5 and H7 play different roles in FXR activity with these ligands. Finally, we tested whether mutating H5 interferes with OCA binding to FXR. In both WT and F340A FXR, we measured OCA binding by monitoring fluorescence polarization while adding OCA to outcompete CLF. This experiment revealed no difference in the binding affinity of OCA between the WT and mutant (Fig. 3L). We also showed *via* Western blots that the F340A mutation does not affect the expression of FXR in luciferase assays (Fig. S6). In summary, H5 mutations do not have an observable impact on dimerization or ligand binding but may weakly impact the interaction with coregulators. In contrast, the H7 mutation noticeably disrupts the recruitment of SRC1-2 to FXR.

### OCA selectively enhances coupling between helix 5 and helix 7

To probe the origin of differences in H5 and H7 mutation effects on FXR activity, we used MD simulations to compare the dynamic behaviors of FXR ligands. We quantified interactions between residues that constitute the FXR “activation trigger,” that is, Y365 on H7, H451 on H10, and W473 on H12 (Fig. S7*A*) (32). We observed a larger distance between W473 and the bile acid A-ring in OCA than CDCA and LCA (Fig. S7*B*). This trend is present in all H5 and H7 mutants, with distances in OCA complexes significantly higher than in the other ligands (Fig. S8*A*). We quantified the occupancy of the hydrogen bond between the Y365 side chain and the A-ring OH, observing that it is significantly reduced in OCA complexes (Figs. S7*C* and S8*B*). We also saw higher distances between the aromatic rings of W473 and H451 in OCA complexes than other ligands (Fig. S7*D*). These observations suggest that OCA attains a unique orientation in the binding pocket compared to the other bile acids, shifting both inter-residue interactions and FXR-ligand contacts.

**Figure 4:**
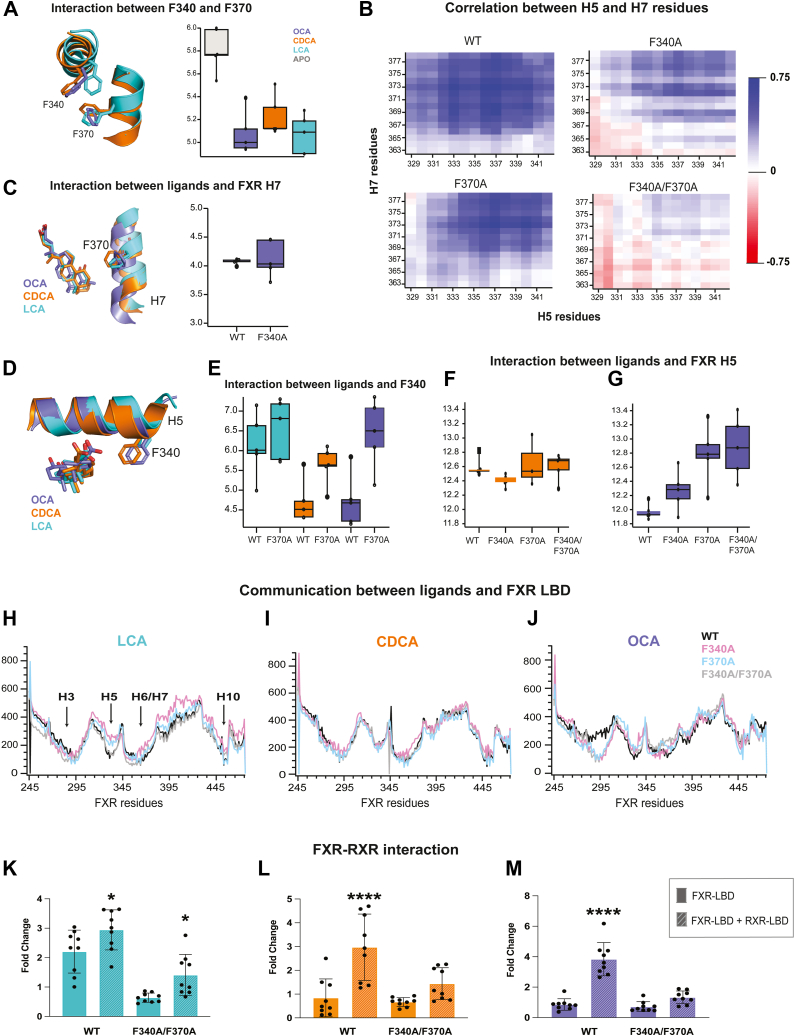
**Helices 5 and 7 are coupled in FXR LBD.***A*, Pi-stacking interactions between F340 and F370 in APO FXR and ligand-bound complexes. *B*, correlation plots between H5 and H7 residues in WT, F340A, F370A, and F340A/F370A complexes in the absence of ligand. *C*, interaction of ligands with H7 in WT and F340A complexes. *D–G*, ligand interactions with F340 and H5 in WT and mutant complexes. *H–J*, shortest distance analysis depicting communication patterns between ligands and FXR residues. The wells in the curves identify regions with the lowest shortest distance values (H3, H5, H6/H7, and H10), which serve as conduits for communication between the ligand binding pocket and the LBD. Communication is compared in WT FXR, H5, and H7 single and double mutants. While LCA and CDCA display similar patterns in communication, OCA displays a notable difference in the WT FXR complex, with the loss of the H3 well. This suggests that OCA preferentially induces allosteric communication through alternate conduits in the WT receptor. Trajectory data from (*A*) to (*J*) represent data obtained from five 500-ns replicates of all FXR–ligand complexes. *K–M*, luciferase assays testing the interaction between WT and double mutant (F340A/F370A) constructs with RXR-LBD. Here we observe that the double mutant disrupts the interaction between FXR and RXR in OCA- and CDCA-treated cells. CDCA, chenodeoxycholic acid; FXR, farnesoid X receptor; LBD, ligand-binding domain; OCA, obeticholic acid; RXR, retinoid X receptor.

We also observed that due to the proximity of H5 and H7, F340 and F370 side chains are positioned for pi-stacking (Fig. 4*A*). In the absence of ligand, the two phenyl rings are ∼5.8 Å apart. The addition of any of the ligands decreases the distance to 5 to 5.2 Å, suggesting a role for the ligand in stabilizing the putative pi–pi interaction. Based on this observation, we hypothesized that H5 and H7 are functionally and dynamically coupled, that is, the motions of H5 can influence the function and dynamics of H7, and *vice versa*. To test this hypothesis, we first obtained MD trajectories on the double F340A/F370A mutant. We used a covariance analysis to compare the correlation between H5 and H7 in the WT, F340A, F370A, and F340A/F370A FXR constructs (Fig. 4*B*). In the absence of ligand, we observed a positive correlation between both helices in WT FXR, supporting the hypothesis of coupling. The addition of mutations progressively reduced this correlation, with extensive anticorrelation observed in F340A/F370A, suggesting that F340A and F370A disrupt H5-H7 coupling. In the presence of ligands, we observed weaker correlation across all complexes and not much difference between the mutant and WT forms.

To understand how the H7 mutation affects H5, we quantified the interaction between ligands and H5 in both WT and F370A (H7 mutant) FXR constructs (Fig. 4*D*). Both CDCA and OCA contact F340 (distance <4.5 Å) in WT FXR, while LCA does not. For both ligands, the H7 F370A mutation abolishes contact with F340 on H5, providing support for coupling between the two helices (Fig. 4*E*). To quantify the proximity of the ligand to the entire helix, we measured the distance between OCA and CDCA and the center of mass of H5 (Fig. 4, *F* and *G*). In OCA-bound complexes, both H5 and H7 mutations reduce the proximity between H5 and the ligand. The F340A/F370A double mutant shows a synergistic increase in the separation, supporting the coupling between the residues (Fig. 4*G*). This increased separation from H5 is not observed in CDCA complexes when mutations are introduced (Fig. 4*F*), suggesting that OCA strengthens the coupling between H5 and H7 in a unique manner absent in other ligands. We also quantified the interaction between the ligands and H7 (Fig. 4*C*). As expected, only OCA contacts F370, mediated by the C6 ethyl group. However, this contact is not impaired in the H5 (F340A) mutant, in contrast to the observed separation between ligands and H5 that was induced by mutating H7. This observation suggests that in the coupling between the two helices, mutating H7 is more consequential on ligand interactions than mutating H5. We note that this finding is seemingly consistent with experimental results where F370A (H7 mutant) showed a larger effect than F340A (H5 mutant) on FXR–SRC1-2 interaction.

Finally, to describe communication patterns between the ligands and FXR residues, as well as to understand how this relates to the coupling between H5 and H7, we used a shortest distance analysis (Fig. 4, *H–J*). This analysis reveals a striking difference between OCA and the other ligands with regard to signaling patterns. Shortest distance plots have four minima (or wells) located at residues 290 to 295, 330 to 335, 355 to 365, and 450 to 455. These wells identify H3, H5, H6, and H10, respectively, as the conduits for communication from the ligand binding pocket to the rest of the receptor. In both LCA and CDCA, we observed that H5 and H7 mutations do not alter these predicted communication patterns (Fig. 4, *E* and *I*). Unexpectedly, the H3 well is absent in the WT OCA complex, suggesting that OCA excludes H3 for communication, likely preferring H5 instead (Fig. 4*J*). By enhancing the coupling between H5 and H7, OCA destabilizes H3-mediated communication and forces signaling through H5 and other helices. Mutation of H5 and H7, which breaks H5-H7 coupling, also restores the H3 well in the shortest distance analysis, allowing OCA to revert to the use of H3 for communication, as is observed in LCA and CDCA.

Based on these combined observations, we hypothesized that the double H5/H7 FXR mutant would reveal impaired function in our previously described M2H assays. The F340/F370 FXR mutant did not affect the interaction with PGC-1α, compared to WT FXR (Fig. S4*C*). However, a significantly reduced interaction is observed with the SRC1-2 peptide, similar to the effect seen with the H7 mutation alone in OCA and CDCA-treated cells (Fig. S4*D*). In contrast, the double mutant inhibits FXR-RXR heterodimer formation when treated with OCA and CDCA, which was not observed in either individual mutant (Fig. 4, *K–M*). To summarize, the double mutation exerts a synergistic effect on FXR, specifically impacting dimerization with RXR.

### H5-H7 coupling mechanism is not conserved in ligand with alternate scaffold

**Figure 5:**
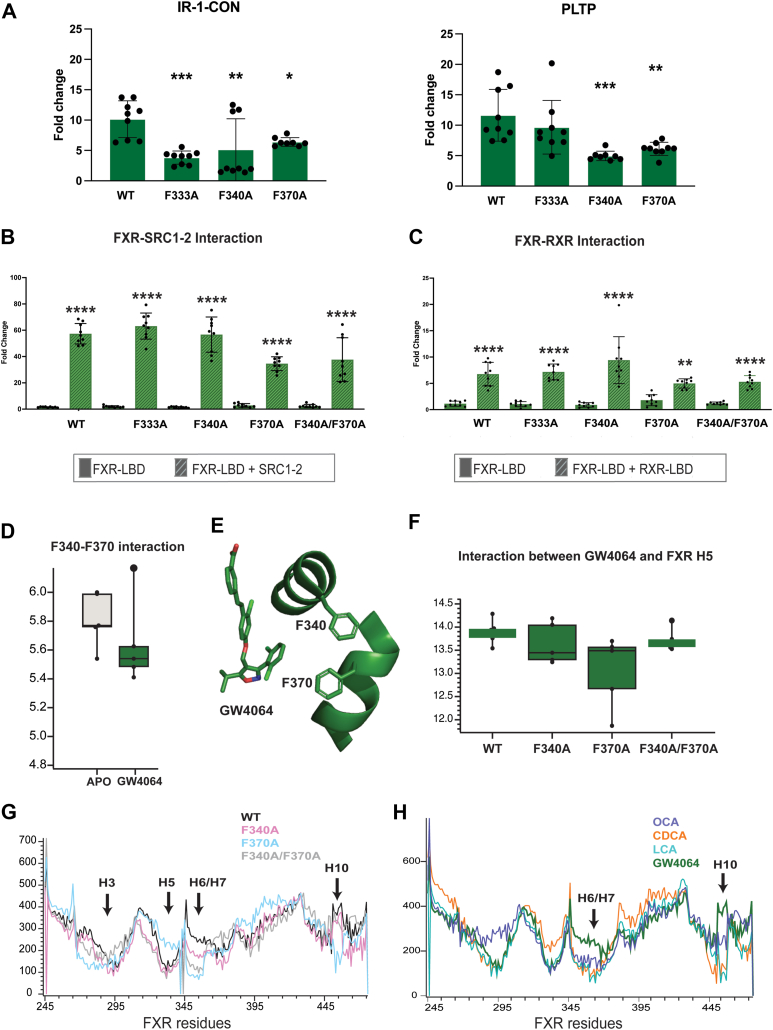
**GW4064 modulates FXR activity differently from bile acids.***A*, luciferase assays comparing GW4064 activity at 10 μM in WT FXR and H5 and H7 mutants. Both IR-1-CON-*luc* and PLTP-*luc* reporters are used to assay transcriptional activity. Mutations reduce the activity of GW4064. *B*, M2H luciferase assay testing the interaction between WT and mutant (F333A, F340A, F370A, and F340A/F370A) FXR constructs with SRC1-2 coregulator in the presence of 10 μM GW4064. The interaction is not impaired by FXR mutations. *C*, M2H luciferase assay testing the interaction between WT and mutant (F333A, F340A, F370A, and F340A/F370A) FXR constructs with RXR, in the presence of 10 μM GW4064. The interaction is not impaired by the mutations. *D*, interaction between H5 and H7 measured by the distance between side chains of F340 and F370. Comparison between APO and GW4064-bound FXR shows that GW4064 only exerts a small influence on the distance, compared to that observed in bile acids. *E*, GW4064 is not within contact with H5 and H7. *F*, distance between GW4064 and H5 is compared across all four complexes. No effect on the distance is observed in H5 or H7 mutants. *G*, shortest distance analysis to quantify communication between the ligand and the entire FXR LBD reveals four wells (H3, H5, H6/H7, and H10), similar to bile acid complexes, representing conduits of communication between ligand binding pocket and the LBD. *H*, comparison of bile acids with GW4064 ligand communication in WT FXR reveals some differences, primarily at H6/H7 and H10. Trajectory data from (*D*) to (*H*) represent data obtained from five 500-ns replicates of all FXR–ligand complexes. FXR, farnesoid X receptor; LBD, ligand-binding domain; M2H, mammalian two-hybrid system; RXR, retinoid X receptor; SRC, steroid receptor coactivator.

The discovery of H5-H7 coupling when FXR LBD is bound to bile acids motivated us to study the ligand dependence of this mechanism. Using the synthetic agonist GW4064 (EC50 = 75 nM) (53) with a nonbile acid scaffold, we assessed whether similar trends to OCA would be observed, suggesting that all potent FXR ligands uniquely modulate H5 and H7. Through dual luciferase assays, we demonstrated that H5 and H7 mutations significantly reduced GW4064 transactivation (Fig. 5*A*), similar to observations with OCA (Figs. 2*D* and S2). We note that the magnitude of this reduction is higher in OCA than GW4064. The FXR–PGC-1α interaction in response to GW4064, quantified by the M2H assay, was not affected by H5 or H7 mutations (Fig. S9). This was also consistent with the behavior of bile acids. In contrast, while the H7 mutant disrupted the interaction with SRC1-2 with bile acids, there was no effect of the H7 mutation in GW4064-treated cells (Fig. 5*B*). Further, although the double H5/H7 mutation disrupted FXR-RXR heterodimerization by bile acids, this activity was not affected in the GW4064 complex (Fig. 5*C*). Thus, GW4064 seemingly modulates FXR activity through a distinct mechanism compared to bile acids.

We also used MD simulations to study GW4064 complexes. Unlike in bile acids, which reduced the F340-F370 distance to ∼5.2 Å, the addition of GW4064 only reduced the distance to ∼5.5 Å, suggesting that this ligand does not stabilize pi-stacking between H5 and H7 to the same extent (Fig. 5*D*). GW4064 does not contact either F340 (H5) or F370 (H7) in WT FXR (distance 7.5 Å), potentially explaining why it does not contribute to the coupling between the helices (Fig. 5*E*). This lack of interaction persists in the mutant complexes (Fig. 5*F*). Finally, we used a shortest distance analysis to characterize communication between GW4064 and the LBD (Fig. 5*G*). Minima are observed in the same four regions (H3, H5, H7, and H10/H11), suggesting that similar residues are used as conduits for communication. Some differences can be observed between GW4064 and bile acids (Fig. 5*H*). First, GW4064 excludes the intervening H6-H7 loop from the H6-H7 conduit, supporting a differential engagement with H7 than bile acids. Distances to H6 are shorter than H7, indicating a bias toward interacting with H6, whereas bile acids display equal communication to H6, H7, and the loop. Additionally, shortest distances to H10 are weaker than to H3, H5, and H6, suggesting attenuated signaling through this avenue. In summary, our experiments with GW4064 support the conclusion that while OCA likely achieves potent activity by enhancing coupling between H5 and H7, GW4064 uses a different mechanism.

## Discussion

Modifications to the scaffolds of nuclear receptor ligands are a common way to enhance their activity (54, 55, 56). These modifications can alter activity by a variety of mechanisms, ranging from improving binding affinity to reshaping the AF-2 surface to modulate coregulator recruitment (41). Here, we aim to understand how the C6 ethyl group in OCA achieves a 100-fold increase in potency over the endogenous agonist CDCA in FXR activation. Both OCA and CDCA exhibited nearly equivalent effects on coregulator recruitment and dimerization (35), suggesting a different underlying mechanism. Our binding assay disproved prior speculations (46) that the increase in activity resulted from enhanced binding of OCA to FXR. Taking the approach of probing ligand-induced dynamics in FXR, we identified H5 as a region whose dynamics are uniquely perturbed in FXR-OCA. This connection was not intuitive, as all three bile acids in this study have the same chemical structure in the region that faces H5. By combining simulations, mutational analysis, and functional assays, we reveal that the dynamic effect observed in H5 stems from its coupling with H7. Because the ethyl tail of OCA contacts H7, this ligand is uniquely capable of strengthening the coupling between H5 and H7.

**Figure 6:**
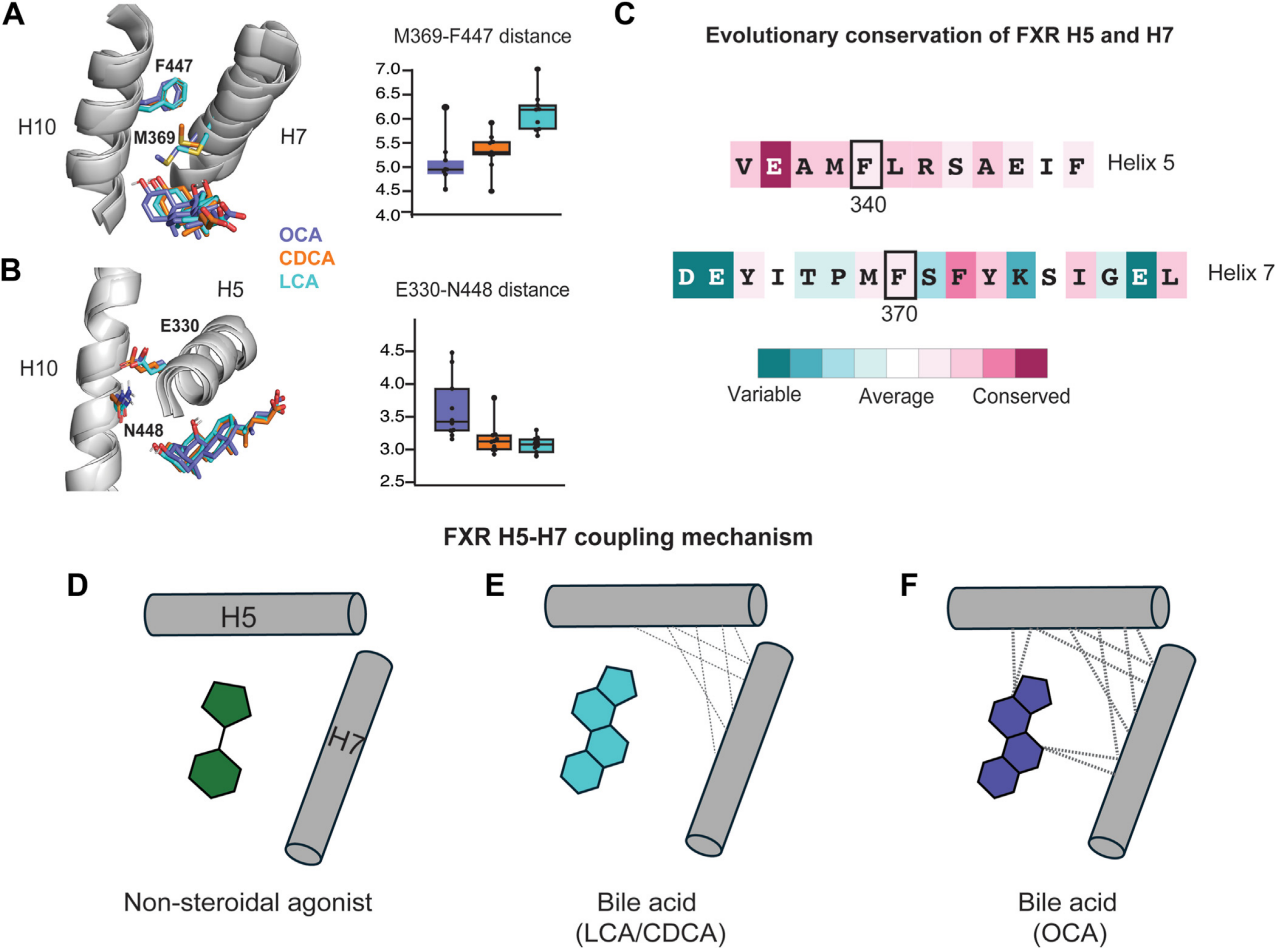
**Unique mechanism of activation by bile acids.***A*, the H7-H10 interhelical distance (M369-F447) is reduced in OCA complexes, indicating a stronger interaction between the two helices. *B*, distance between the hydrogen bonding pair E330 (H5) and N448 (H10) is quantified. OCA complexes reveal further distances, correlated to lower occupancy of the hydrogen bond. *C*, ConSurf analysis of evolutionary conservation of FXR H5 and H7 among 300 nuclear receptors primarily from the NR1 subfamily. Residues F340 and F370 are highlighted on both helices, indicated as moderately conserved. Overall, H5 is more conserved than H7. *D–E*, comparison of the putative H5-H7 FXR mechanism across three types of ligands. *D*, in a nonsteroidal FXR agonist (*e.g.*, GW4064), the FXR H5-H7 coupling mechanism is nonexistent (or weak). *E*, in lower potency agonists, for example, LCA or CDCA, H5 and H7 dynamics are coupled. *F*, OCA, a potent bile acid with C6 alkyl substitution, strengthens coupling between H5 and H7 by directly interacting with the helices. CDCA, chenodeoxycholic acid; FXR, farnesoid X receptor; LCA, lithocholic acid; OCA, obeticholic acid.

Our results identify an important role for H5 and H7 in FXR, as the double mutant (F340A/F370A) impacted both the SRC1-2 interaction and heterodimer formation. We speculated that this effect could arise from the proximity between H7 and H10, which is the dimerization interface of FXR. Even though the F370 side chain is not oriented towards H10, the distance between neighboring residue M369 (H7) and F447 (H10) is significantly lower in OCA complexes (Fig. 6*A*). Thus, the contact between OCA and H7 appears to directly influence H10 behavior. H10 may also be influenced by H5 dynamics, as our simulations show that hydrogen bonding between the helices (E330-N448) is reduced in OCA complexes (Fig. 6*B*). Thus, simulations suggest multiple plausible routes by which H5-H7 coupling in FXR can influence H10. Interestingly, this reduction is not observed when a nonbile acid ligand is bound, confirming that this mechanism of H5-H7 coupling is unique to bile acids.

We note that H5 and H7 play important roles in several nuclear receptors. In RXR heterodimers, H5 is critical for the transmission of allosteric signals, a mechanism conserved in many receptors. H5 of RXR integrates peroxisome proliferator-activated receptor gamma ligand-induced allosteric signals from the dimer interface to RXR ligand binding pocket and the AF-2 surface (57). Similarly, thyroid hormone receptor-induced rotation of RXR H11 leads to a corresponding rotation of RXR H5, which silences RXR activity by affecting the conformation of the AF-2 surface (57). Furthermore, RXR-induced rotation of LXR H5 stabilizes LXR H12 in the active conformation. In steroid receptors, H5 collaborates with H3 to regulate hormone selectivity (38, 58). H7 is shown to be essential for HNF4α homodimerization, transcriptional activity, and recruitment of coactivators (59). A role was also established for H7 of liver receptor homolog-1 in allosterically regulating communication and coregulator recruitment (60). Thus, the identification of H5 and H7 in our study follows existing precedents in nuclear receptors. However, a Consurf analysis characterizes F340 and F370 as both moderately conserved (Fig. 6*C*) in an alignment of over 300 NR1 subfamily receptors across taxa (61, 62, 63). Overall, H5 is more conserved than H7, suggesting that the coupling mechanism is specific to FXR.

In summary, our work illustrates the complexity of ligand-induced dynamics in nuclear receptors. It is clear that bile acids modulate these dynamics differently than other ligand scaffolds, inducing the H5-H7 coupling that is revealed through our simulations (Fig. 6, *D* and *E*). Other previously identified mechanisms in synthetic FXR ligands include fluctuations in H8^40^. Our rigorous MD-based approach reveals that the potency of OCA in FXR is mediated through its dynamic effects on H5, which are linked to the interaction between the 6-ethyl group and H7 of FXR. By strengthening H5-H7 coupling, OCA rewires allosteric communication from the ligand binding pocket, causing signals to preferentially travel through H5 instead of H3. While bile acids induce H5-H7 coupling, we propose a triangulated mechanism whereby OCA strengthens this coupling *via* direct interaction with H7 (Fig. 6*F*). We note that the insight from this study is obtained based only on three bile acids, and that a broader structure-activity relationship analysis of the bile acid scaffold would potentially produce deeper insight into the proposed H5-H7 coupling mechanism. Additionally, these experiments describe ligand activity by measuring efficacy rather than potency. We anticipate that the mechanisms proposed here underlie both OCA efficacy and potency, but additional work would be required to dissect this hypothesis. While the importance of H5/H7 dynamics in FXR is still not fully understood, our results hint at an impact on the FXR–RXR interaction and the FXR–SRC1-2 interaction. Further work would be required to understand why OCA’s ability to modulate H5 dynamics results in increased activity. Moreover, enhancing coupling between H5 and H7 likely represents one way that FXR ligands can achieve potent activity in the receptor, begging the question of whether any nonbile acid FXR ligands can also co-opt this H5-H7 coupling mechanism. It is also worth exploring whether the H5–H7 axis can be used in the design of selective FXR modulators.

## Experimental procedures

### Cloning/preparation of constructs

Human FXRɑ (NR1H4, UniProt Q96RI1-1) and RXRɑ (NR2B1, UniProt P19793-1) were cloned into the pcDNA3.1(+) expression vector, using forward and reverse primers containing EcoRI and HindIII restriction sites. To create constructs for luciferase reporter assays, we modified pGL4.31[*luc2P*/*Gal4*UAS/Hygro] by inserting two copies of the FXR response element (FXRE) and the minimal promoter upstream of the firefly luciferase gene. pGL4.31 was first digested by Dpn1-HF and HindIII-HF, followed by PCR amplification with primers minP_FWD (to append the minimal promoter) and pgl4_REV. Next, two copies of the FXRE sequences IR-1-CON and PLTP were inserted using Gibson assembly.

For protein expression and purification, the LBD of FXR (residues 247–476) was cloned into the expression vector pMalCH10T containing a histidine tag and the maltose-binding protein (MBP). The LBD was inserted upstream of the histidine and MBP tags. Both the expression vector and insert were linearized and amplified (see primer sequences below), followed by Gibson assembly.

Constructs for the mammalian-two-hybrid protein–protein interaction assay were mostly synthesized by GenScript. The FXR-LBD (residues 247–476) was cloned into the pACT plasmid (Promega) to generate fusion proteins with the VP16 activation domain to give VP16-(FXR-LBD). The PGC-1α (residues 100–411) was cloned into the mammalian Gal4DBD fusion vector pBIND (Promega) to give Gal4DBD-(PGC-1α). The SRC1-2 construct was made by inserting these residues (LTERHKILHRLLQEGSPSD) on the N-terminal end of the Gal4DBD. RXR-LBD (residues 223–462) was cloned into the pBIND vector to give Gal4DBD-(RXR-LBD) using Gibson assembly.

List of primers

**Table:** 

Primers	Sequence
FXR PCDNA3.1_FWD	5′ GCTTGGTACCATGGGATCAAAAATGAATCTCATT 3′
FXR PCDNA3.1_REV	5′ AACCCTCGAGCTGCACGTCCCAGATTTC 3′
RXR PCDNA3.1_FWD	5′ TGCTGGAATTCATGGACACCAAACATTTC 3′
RXR PCDNA3.1_REV	5′ GCCCTCTAGACAAAGTCATTTGGTGCGG 3′
minP_FWD	5′ AGACACTAGAGGGTATATAATGGAAGCTCGACTTCCAG CTTGGCAATCCGGTACTGT 3′
pgl4_REV	5′ CGGTACCGGCCAGTTAGGCC 3′
IR-1-CON_insert	5′AACTGGCCGGTACCGTCAAG**AGGTCATTGACCT**TTTTGTCAAG**AGGTCATTGACCT**TTTTGAGACACTAGAGGGT 3′
PLTP_insert	5′AACTGGCCGGTACCGCAACTGA**GGGTCAGTGACCC**AAGTGACAACTGA**GGGTCAGTGACCC**AAGTGAAGACACTAGAGGGTA 3′
pMALCH10t_FWD	5′ GGACGTGCAGTGAAAGCTTGGCCTGG 3′
pMALCH10t_REV	5′ TTTTCAGGGAACTGAACTCACCCCAGATCAAC 3′
FXR LBD_FWD	5′ TTTTCAGGGAACTGAACTCACCCCAGATCAAC 3′
FXR LBD_REV	5′ CAAGCTTTCACTGCACGTCCCAGATTT 3′
RXR_LBD_FWD	5′ CAATGTATCTTATCACAAAGTCATTTGGTGCGGC 3′
RXR_LBD_REV	5′ CAGTTGACTGTATCGACCAGCAGCGCC 3′
pBIND_FWD	5′ CACCAAATGACTTTGTGATAAGATACATTGATGAGTTTGGACAAACCACA 3′
pBIND_REV	5′ GTTGGCGCTGCTGGTCGATACAGTCAACTGTCTTTGACCTTTG 3′
M332A_FWD	5′ CAGCTGAACGAAGGAACGCAGCTTCAACCGCAGACC 3′
M332A_REV	5′ GGTCTGCGGTTGAAGCTGCGTTCCTTCGTTCAGCTG 3′
F333A_FWD	5′ AATCTCAGCTGAACGAAGGGCCATAGCTTCAACCGCAGAC 3′
F333A_REV	5′ GTCTGCGGTTGAAGCTATGGCCCTTCGTTCAGCTGAGATT 3′
R335A_FWD	5′ AAATCTCAGCTGAAGCAAGGAACATAGCTTCAACCGCAG 3′
R335A_REV	5′ TCTGCGGTTGAAGCTATGTTTCCTTGCTTCAGCTGAGATTT 3′
F340A_FWD	5′ CCAGACGGAAGTTTCTTATTGGCAATCTCAGCTGAACGAAGGAA 3′
F340A_REV	5′ TTCCTTCGTTCAGCTGAGATTGCCAATAAGAAACTTCCGTCTGG 3′
Y365A_FWD	5′ TTTTATAAAAACTAAACATAGGTGTTATAGCTTCATCAGAGATACCACTATTTCAATTC 3′
Y365A_REV	5′ GAATTCGAATAGTGGTACTCTGATGAAGCTATAACACCTATGTTTAGTTTTTATAAAA 3′
M369A_FWD	5′ GTTCCCCAATACTTTTATAAAAACTAAACGCAGGTGTTATATATTCATCAGAGATACCAC 3′
M369A_REV	5′ GTGGTATCTCTGATGAATATATAACACCTGCGTTTAGTTTTTATAAAAGTATTGGGGAAC 3′
F370A_FWD	5′ TCAGTTCCCCAATACTTTTATAAAAACTAGCCATAGGTGTTATATATTCATCAGAGATAC 3′
F370A_REV	5′ GTATCTCTGATGAATATATAACACCTATGGCTAGTTTTTATAAAAGTATTGGGGAACTGA 3′
Y373F_FWD	5′ CATTTTCAGTTCCCCAATACTTTTAAAAAAACTAAACATAGGTGTTATATATTCATC 3′
Y373F_REV	5′ GATGAATATATAACACCTATGTTTAGTTTTTTTAAAAGTATTGGGGAACTGAAATG 3′

### Protein expression and purification

*Escherichia coli* BL21 cells (NEB) were transformed and grown to an *A*_600_ of 0.5 to 0.6 and then induced with 0.6 mM of IPTG to express the MBP-FXR-LBD fusion protein. For protein purification, cells were lysed with lysis buffer (20 mM Tris pH 8.0, 300 mM NaCl, 10% glycerol, 25 mM imidazole, 2 mM DTT, and 2 mM PMSF) and sonicated. The cellular debris was spun down and separated from the supernatant. The supernatant was applied to the Bio-Rad RAD 5 ml Ni-charged affinity column. The protein was eluted at 32 to 34% buffer B (20 mM Tris pH 8, 300 mM NaCl, 10% glycerol, and 1 M imidazole) within buffer A (20 mM Tris pH 8, 300 mM NaCl, 10% glycerol, 1 mM DTT, and 25 mM imidazole). Afterward, the sample was placed in dialysis tubing inside dialysis buffer (20 mM Tris pH 8, 150 mM NaCl, 10% glycerol, and 1 mM DTT) at 4 °C overnight while spinning. The following day, size-exclusion chromatography was performed using an ENrich SEC 650column (Bio-Rad). After size exclusion, the sample was concentrated using a 3 K molecular weight cut-off Pierce concentrator (Thermo Fisher Scientific).

MBP-tagged full-length FXR was expressed and purified similarly to the LBD version discussed above, except with a slight change in buffer conditions (buffer A: 20 mM Tris pH 7.4, 500 mM NaCl, 10% glycerol, 1 mM DTT, 25 mM imidazole, and buffer B: 20 mM Tris pH 7.4, 500 mM NaCl, 10% glycerol, 1 mM DTT, and 500 mM imidazole). The fraction eluted at 250 mM imidazole was subjected to dialysis against buffer (20 mM Tris pH 7.4, 150 mM NaCl, 10% glycerol, and 1 mM DTT) overnight at 4 °C with three buffer changes. The dialyzed sample was concentrated to 6 to 8 mg/ml using 10 KDa Amicon (MilliporeSigma) and further purified by gel-filtration chromatography using ENrich SEC650 column (Bio-Rad). The gel-filtration buffer was 20 mM Tris pH 7.4, 150 mM NaCl, 10% glycerol, and 2 mM DTT. The purified protein sample was flash-frozen in small aliquots with liquid nitrogen and stored at −80 °C until further use.

### Fluorescence polarization competitive ligand binding assay

For the competition assays, a range of OCA, CDCA, and LCA concentrations (from 1 μM to 1000 μM) were used to outcompete 100 nM of CLF. The ligands were dissolved initially in dimethyl sulfoxide (DMSO) in a 20 mM stock and were diluted to a 2 mM working stock using the assay buffer (20 mM Tris pH 8, 150 mM NaCl, 5% glycerol, and 1 mM DTT). Purified FXR (FL or LBD)-MBP was diluted to a final concentration of 20 μM using assay buffer. Samples were set up in triplicate in a 384 black flat-bottom plate and incubated for 30 min before reading on a SpectraMax iD5 plate reader. Assays were performed in triplicate, and the data were combined and fitted to a one site-Fit Ki equation with GraphPad Prism (www.graphpad.com) (version 10.2.3) to determine the Ki. Binding curves for OCA and CDCA data are from three independent replicates, whereas LCA data were from two independent replicates.

### Luciferase assays

#### Cell lines and cell culture

HeLa cells were obtained from American Type Culture Collection, and the cells were grown in culture in minimum essential medium alpha (Thermo Fisher Scientific) supplemented with 10% FBS and 1% L-glutamine at 37 °C with humidified atmosphere at 5% CO_2_.

#### HeLa transfection and dual-luciferase assay

Cells were seeded 10,000 cells/well in a 96-well, clear flat, bottom cell culture plate. Cotransfection was performed with equal amount of 5 ng of each expression plasmids pcDNA3.1-FXR and pcDNA3.1-RXR, along with 50 ng of the pGL4.31-derived plasmids containing the FXRE and 1 ng of renilla luciferase (pRL-CMV). Two different IR-1 (inverted repeat spaced by one nucleotide) FXREs were used, the IR-1 consensus sequence (AGGTCAtTGACCT) and PLTP IR-1 sequence (GGGTCAgTGACCC). Each FXRE plasmid construct contained two copies of either the IR-1-CON sequence or the PLTP IR-1 sequence. Transfection was repeated at least three times with Fugene HD (Promega). In coregulator studies, plasmids containing pSG5-SRC-1, pDEST26-PGC-1α, or pDEST26-SRC-3 were also transfected. These coregulator plasmids were gifts from Dr Eric Ortlund (Emory University).

At 24 h after transfection, cells were stimulated with DMSO (final concentration of 0.71%) or test ligands at final concentrations 10 μM or 100 μM, as appropriate. After another 24 h incubation, Firefly luciferase activity and Renilla luciferase activity were measured using the Dual-Glo kit (Promega) using a SpectraMax iD5 plate reader. Fold activation was represented as normalized luciferase over DMSO-treated controls, which were treated with 0.71% DMSO in all conditions.

Data for luciferase assays are presented as the mean ± standard SD of at least three independent replicates. *p* values were calculated using two-way ANOVA with Dunnett’s multiple comparisons test to access the differences between specific pairs of means. For coregulator mean comparisons, *p* values were calculated using one-way ANOVA with Sidaks’s multiple comparisons. Significance levels are indicated as follows: *p* < 0.1234 (ns), *p* < 0.0332 (∗), *p* < 0.0021 (∗∗), *p* < 0.0002 (∗∗∗), and *p* < 0.0001 (∗∗∗∗). All statistical analyses were performed using GraphPad Prism (version 10.3.0) software.

#### M2H protein–protein interaction assay

Assays were performed following the instructions of the CheckMate M2H System (Promega). Transient transfection was performed with equal amount of 5 ng of each pBIND and pACT constructs, along with the 50 ng reporter plasmid pG5*luc*, which contains the upstream activating sequence response element and encodes firefly luciferases. Controls included wells with empty pACT vector, VP16 control. Stimulation of cells, measurement of luciferase activity, and data analysis were performed as described in the HeLa transfection and dual-luciferase assay section.

### Mutagenesis

FXR-LBD mutants were generated by PCR-based site-directed mutagenesis with the primers listed.

### Western blotting

HeLa cells were maintained in minimum essential medium alpha and 10% FBS at 37 °C. For transfection, 1 × 10^–6^ cells were seeded in a 6-well plate. After 24 h, 2 μg DNA from either FXR WT or mutant plasmids were transfected into different wells of the plate with Fugene HD (Promega). The cells were then grown for 48 h before being harvested and lysed in a RIPA buffer (50 mM Tris, pH 8.0, 150 mM NaCl, 0.1% Triton X-100, 0.5% sodium deoxycholate, and 1 mM sodium azide and protease inhibitor from Thermo Fisher Scientific) by incubating them on ice for 30 min. The cell lysates were cleared by centrifugation at 12,000 rpm for 30 min at 4 °C. The protein concentration was determined by comparing it with a standard plot of bovine serum albumin and estimated using Bradford reagent (Bio-Rad). Equal amounts (40 μg) of total protein samples were electrophoresed on a 12% SDS-PAGE and transferred to a polyvinylidene fluoride membrane. FXR proteins were detected using ECL (Thermo Fisher Scientific) after incubating with the mouse monoclonal anti-FXR (D-3) antibody (sc-25309, Santacruz Biotechnology) and a horseradish peroxidase–linked secondary antibody (sc-525409, Santacruz Biotechnology).

### MD simulations

#### Model preparation

Structural models of the bile acids were either obtained from the PDB or constructed using the Maestro software suite (https://www.schrodinger.com/platform/products/maestro/) (64). All FXR complexes were constructed using PDB 6HL1, (34) (Human FXR-CDCA with bound NCoA-2 peptide) as a template. All waters and surface-bound molecules from crystallization buffer and the coregulator fragment were deleted, leaving only FXR and CDCA. Missing residues (339–344) were filled in with Modeller (65). To obtain OCA and LCA complexes, the steroidal scaffold on CDCA was modified. To obtain complexes with SRC3-1 and PGC-1α the amino acid sequence of NcoA-2 (SRC-2) (KENALLRYLLDKD) was mutated to the amino acid sequences for SRC3-1(KGHKKLLQLLTCS) and PGC-1α (PSLLKKLLLAPA). Our FXR-OCA model superimposed with the *Rattus norvegicus* FXR-OCA structure (PDB 1OSV) (32) with RMSD <1 Å. Mutant complexes were created by introducing mutations *in silico* using xleap in AmberTools 18 (66). A summary of all the complexes simulated is provided below.

Summary of complexes simulated

**Table:** 

	Ligand	Simulation length	# Replicates
FXR–ligand complexes			
WT FXR	OCA, LCA, CDCA	500–1000 ns	8 replicates
	GW4064	500 ns	5 replicates
	Apo	500 ns	5 replicates
F340A	OCA, LCA, CDCA	500–1000 ns	8 replicates
	GW4064	500 ns	5 replicates
	Apo	500 ns	5 replicates
F370A	OCA, LCA, CDCA	500–1000 ns	8 replicates
	GW4064	500 ns	5 replicates
	Apo	500 ns	5 replicates
F340A/F370A	OCA, LCA, CDCA	500 ns	5 replicates
	GW4064	500 ns	5 replicates
	Apo	500 ns	5 replicates
M332A	OCA, LCA, CDCA	1000 ns	3 replicates
F333A	OCA, LCA, CDCA	1000 ns	3 replicates
R335A	OCA, LCA, CDCA	1000 ns	3 replicates
Y365A	OCA, LCA, CDCA	1000 ns	3 replicates
M369A	OCA, LCA, CDCA	1000 ns	3 replicates
Y373F	OCA, LCA, CDCA	1000 ns	3 replicates
Coregulator complexes			
WT FXR – PGC-1α	OCA, LCA, CDCA	1000 ns	3 replicates
F340A – PGC-1α	OCA, LCA, CDCA	1000 ns	3 replicates
F333A – PGC-1α	OCA, LCA, CDCA	1000 ns	3 replicates
WT FXR – SRC3-1	OCA, LCA, CDCA	1000 ns	3 replicates
F340A – SRC3-1	OCA, LCA, CDCA	1000 ns	3 replicates
F333A – SRC3-1	OCA, LCA, CDCA	1000 ns	3 replicates
WT FXR – SRC-2	OCA, LCA, CDCA	1000 ns	3 replicates
F340A – SRC-2	OCA, LCA, CDCA	1000 ns	3 replicates
F333A – SRC-2	OCA, LCA, CDCA	1000 ns	3 replicates

#### MD simulations

The complexes were solvated in an octahedral box of TIP3P water with a 10 Å buffer around the protein. Na+ and Cl− ions were added to neutralize the protein to the physiological ionic concentration of 150 mM. The systems were set up using Xleap in AmberTools 18 (66), contained in Amber 2018 (67). Parameters for the bile acids were created using Antechamber (68) with the Generalized Amber Forcefield (69). Minimization was performed sequentially with the following sets of restraints: i) 500-kcal/mol.A^2^ restraints on protein and ligand atoms, ii) 100-kcal/mol.A^2^ restraints on protein and ligand atoms, iii) 100-kcal/mol.A^2^ restraints on the ligand atoms, and iv) no restraints on any atoms. The complexes were heated from 0 to 300K, 100 ps run with 10-kcal/mol·A^2^ restraints on all protein and ligand atoms. MD equilibration was performed in the following stages: i) 10 ns with 10-kcal/mol.A^2^ restraints on all protein and ligand atoms using the NPT ensemble, ii) 10 ns with 1-kcal/mol.A^2^ restraints on protein and ligand atoms, iii) 10 ns with 1-kcal/mol.A^2^ restraints on ligand atoms, and iv) no restraints on any atoms. Restraints were then removed and triplicate runs of 500 ns or 1000 ns production simulations were performed for each system in the NPT ensemble. All simulations were performed using the parmbsc0 forcefield (70).

#### Contact maps and network analysis

Averaging of the replicate simulations and analysis was performed using the CPPTRAJ (66, 71) module in AmberTools (66). The commands “strip” and “trajout” from CPPTRAJ were used to extract solvent atoms and obtain a total of 25,000 frames per 500 ns trajectory for analysis. The “distance” command was used to measure the distance between amino acid pairs.

The NetworkView plugin in VMD (72, 73) and the Carma program (74) were used to produce correlation matrices and dynamic networks for each system. The contact maps were generated by stripping all solvent atoms and leaving the ligand and protein atoms. Each protein residue in the contact map was defined as a node, and the distance between nodes over the simulation was defined as edges. An edge is created if heavy atoms of one node are within 4.5 Å of the heavy atoms from the non-neighboring nodes for 75% throughout the trajectory. We also calculate the parameter “shortest distance,” referring to the shortest distance between two nodes in a network. This distance corresponds to either the weight of the edge between the two nodes or the length of the optimal path between them.

Averaging of the replicate simulations and analysis was performed using the CPPTRAJ (71) module in AmberTools20 (66). The commands “strip” and “trajout” from CPPTRAJ (71) were used to extract solvent atoms and to obtain a total of 50,000 frames from each replicate for analysis. The NetworkView plugin in VMD (72, 73) and the Carma program (74) were used to produce contact maps and dynamic networks for each system. The contact maps were generated by stripping all solvent atoms and leaving the ligand and protein atoms. Each protein residue in the contact map was defined as a node, and the distance between nodes over the simulation was defined as edges. An edge is created if heavy atoms of one node are within 4.5 Å of the heavy atoms from the non-neighboring nodes for 75% throughout the trajectory.

#### Suboptimal paths

Communication between the bile acid and AF-2 surface was described by generating suboptimal paths between these sites using the Floyd–Warshall algorithm (75). Communication paths are drawn as a chain of edges connecting the ligand in the ligand binding pocket as the “source” node and an AF-2 residue as the “sink” node, H12 residue E471. As the correlation and edge weights are inversely correlated, the sum of edges along a path between two nodes becomes lower as the strength of communication increases (76). The optimal path is defined as the path with the lowest sum of edges. And a set of suboptimal paths, along with the optimal path, are thought to convey the greatest amount of communication between two distant nodes. The top 1000 suboptimal paths were analyzed, along with the suboptimal paths within a path length cut-off.

## Data availability

All the data described are contained within the article.

## Supporting information

This article contains supporting information.

## Conflict of interest

The authors declare that they have no conflicts of interest with the contents of this article.
