# Supplementary material for: Enhanced dynamic coupling in a nuclear receptor underlies ligand activity
Source: J Biol Chem. 2024 Dec 14;301(2):108081. doi: 10.1016/j.jbc.2024.108081 (PMC11783427; doi:10.1016/j.jbc.2024.108081)
Supplement: Supplemental Figs. S1–S9 [file mmc1.docx]

**Supporting Information**

Enhanced dynamic coupling in a nuclear receptor underlies ligand activity

Tracy Yu^1^*, Priscilla Villalona^1^*, Sabab Hasan Khan^1^, Noriko Mikeasky^1^, Emily Meinert^1^, Jill Magafas^1^, Thilini Pulahinge^2^, Ameen Bader^2^, C. Denise Okafor^1,2‡^

Department of Biochemistry and Molecular Biology, Pennsylvania State University, University Park, PA, 16802, USA

Department of Chemistry, Pennsylvania State University, University Park, PA, 16802, USA


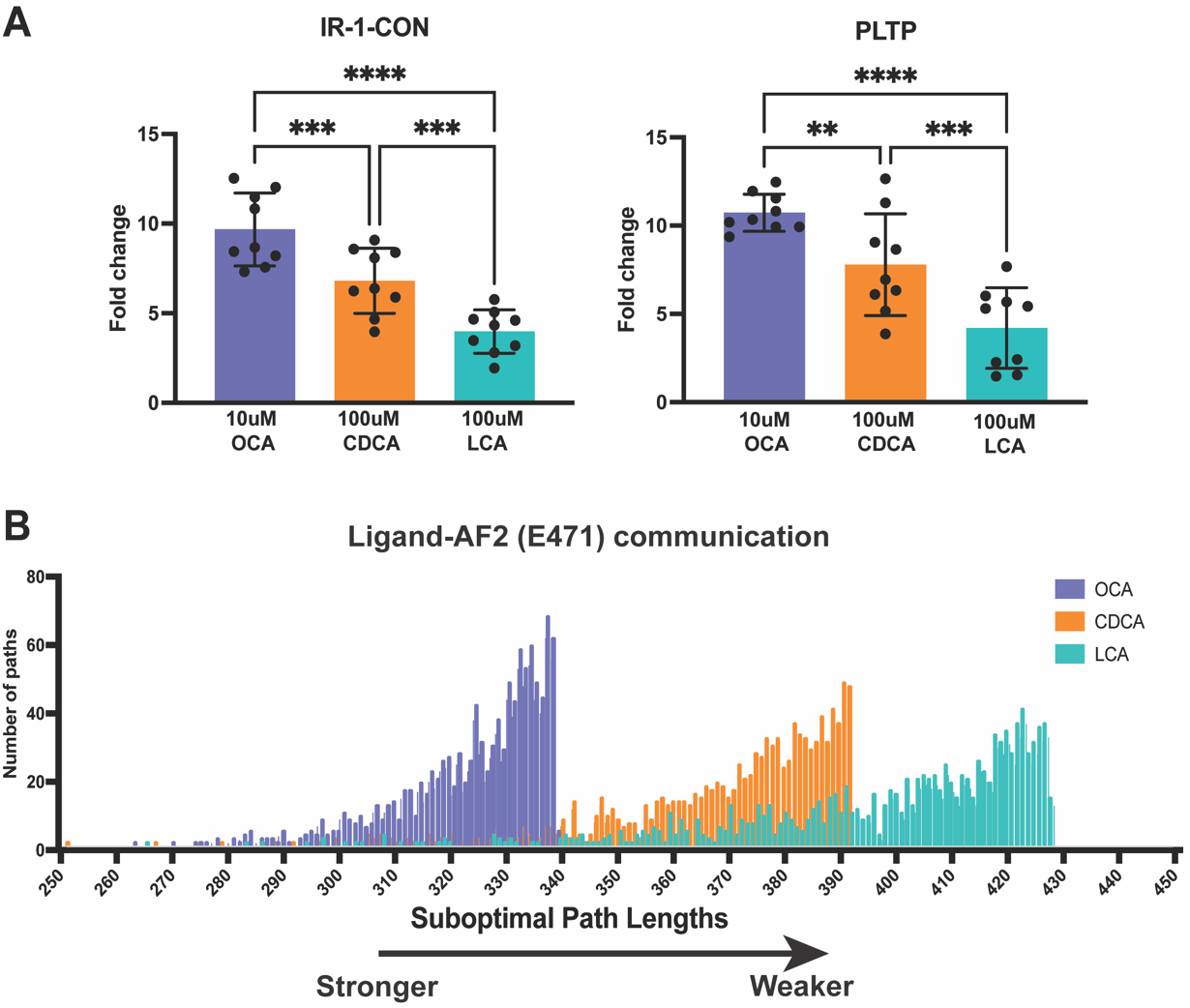


**Figure S1. The activity of OCA, CDCA, and LCA.** **(A)** The activation of OCA in 10 µM is significantly higher than CDCA and LCA at 100 µM in both IR-1-CON-*luc* and PLTP-*luc*. **(B)** Histogram showing the distribution of shortest 1000 paths between the ligand and AF-2 surface residue E471 located on H12 in the WT FXR-LBD. OCA, with the shortest suboptimal path lengths, displays a stronger communication between two sites.


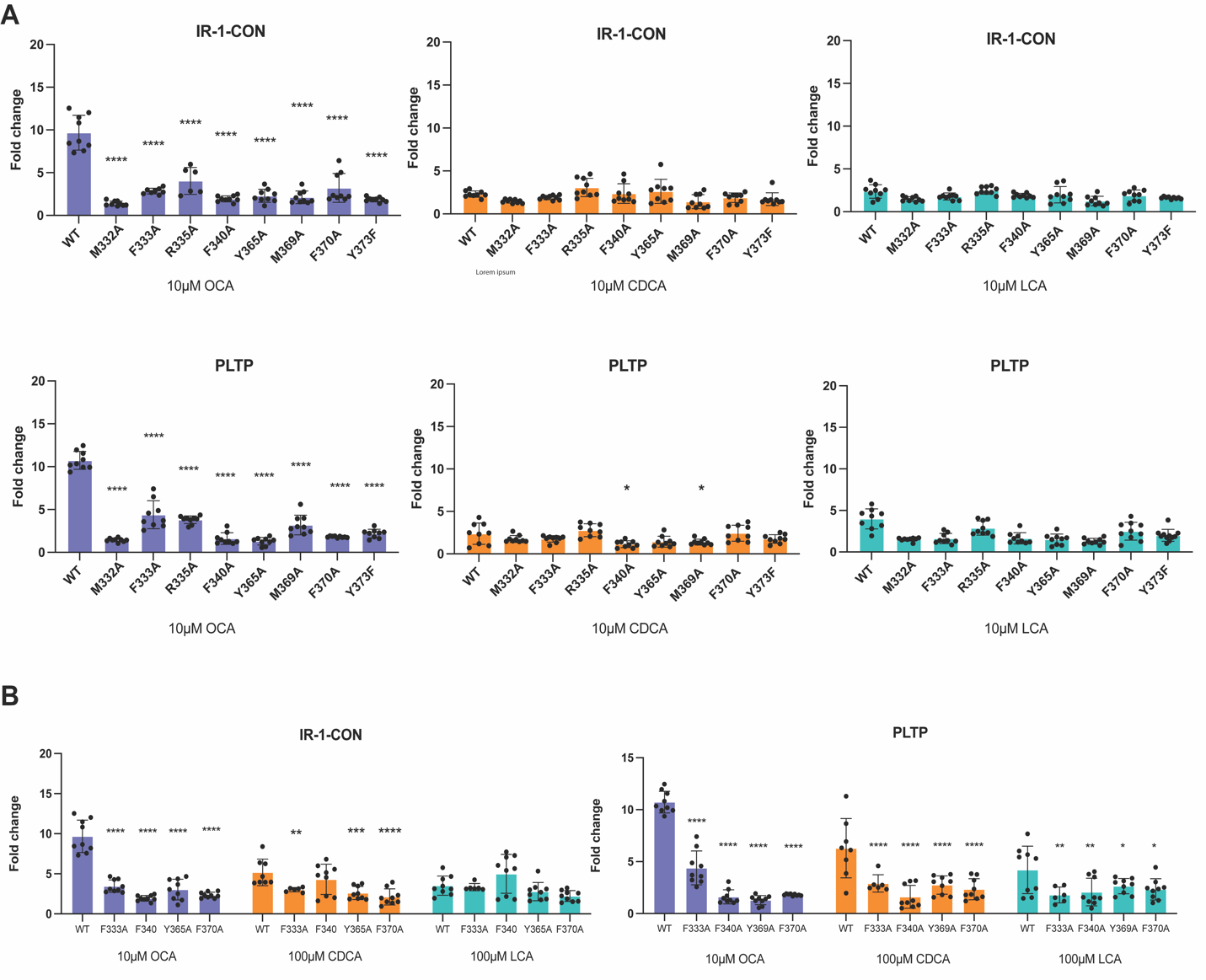


**Figure S2.** **The effect of H5 and H7 mutations on FXR activity.** **(A)** Luciferase activities for H5 and H7 mutants with IR-1-CON-*luc* and PLTP-*luc* at 10 µM bile acids (OCA, CDCA, and LCA). **(B)** Luciferase activities for H5 (F333A and F340A) and H7 mutants (Y365A and F370A) at 100 µM of CDCA and LCA.


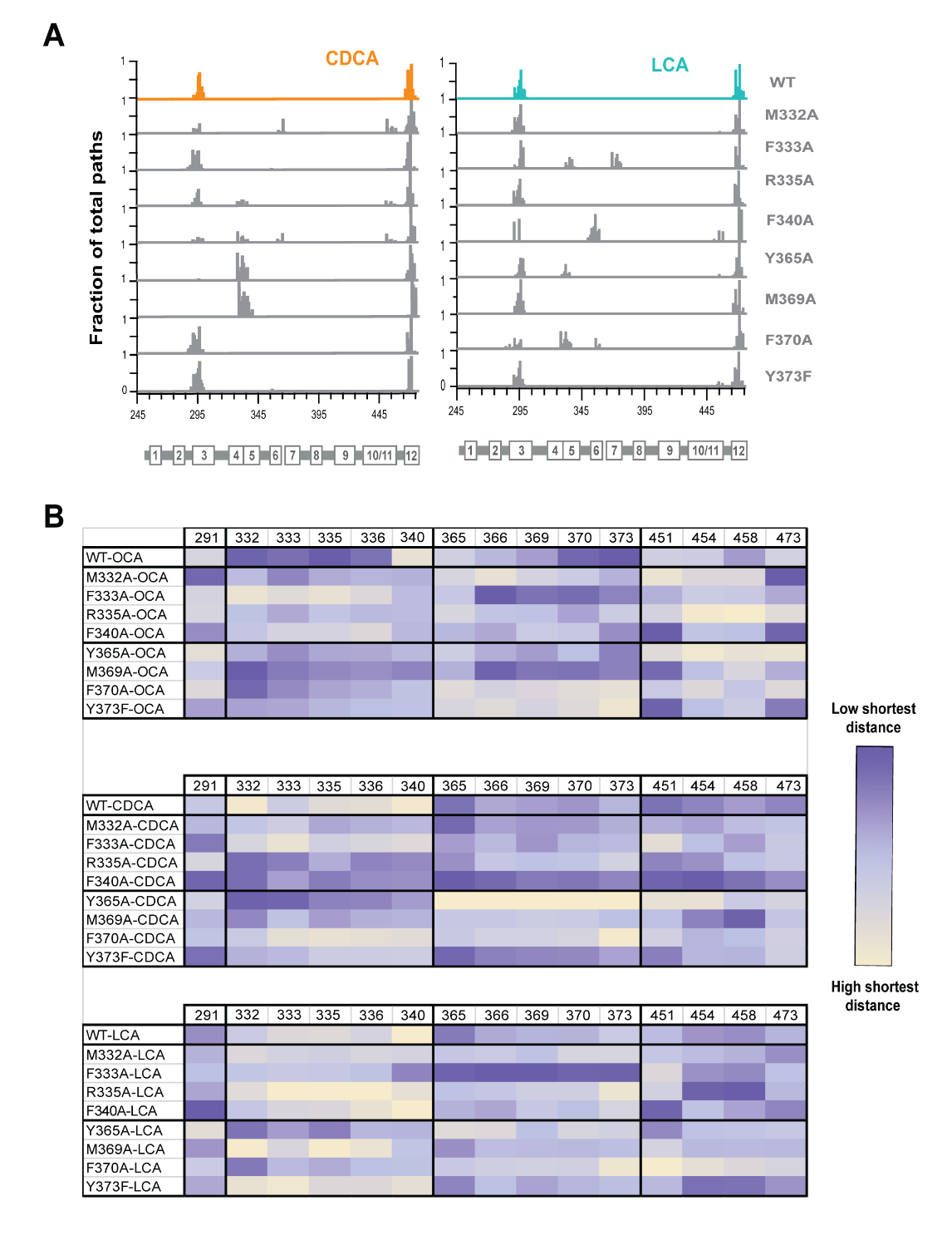


**Figure S3**. **Path length analysis and shortest distance analysis of interactions between bile acid and FXR LBD residues.** **(A)** Histograms show the distribution of the 1000 suboptimal paths of WT, H5, and H7 mutants when interacting with CDCA and LCA, showing that CDCA and LCA do not engage H5 in signaling to AF-2 surface, while several mutants unexpectedly gain H5 involvement. **(B)** Shortest distance analysis between ligands and the surrounding residues. In WT, OCA induces stronger coupling (i.e. smaller shortest distances) with H5 residues, which is mitigated by H5 mutations. F333A and F340A display the highest shortest distances to H5 in OCA complex. Color gradient represents the strength of communication, with darker purple colors indicating shorter distances and lighter beige colors indicating longer distances.


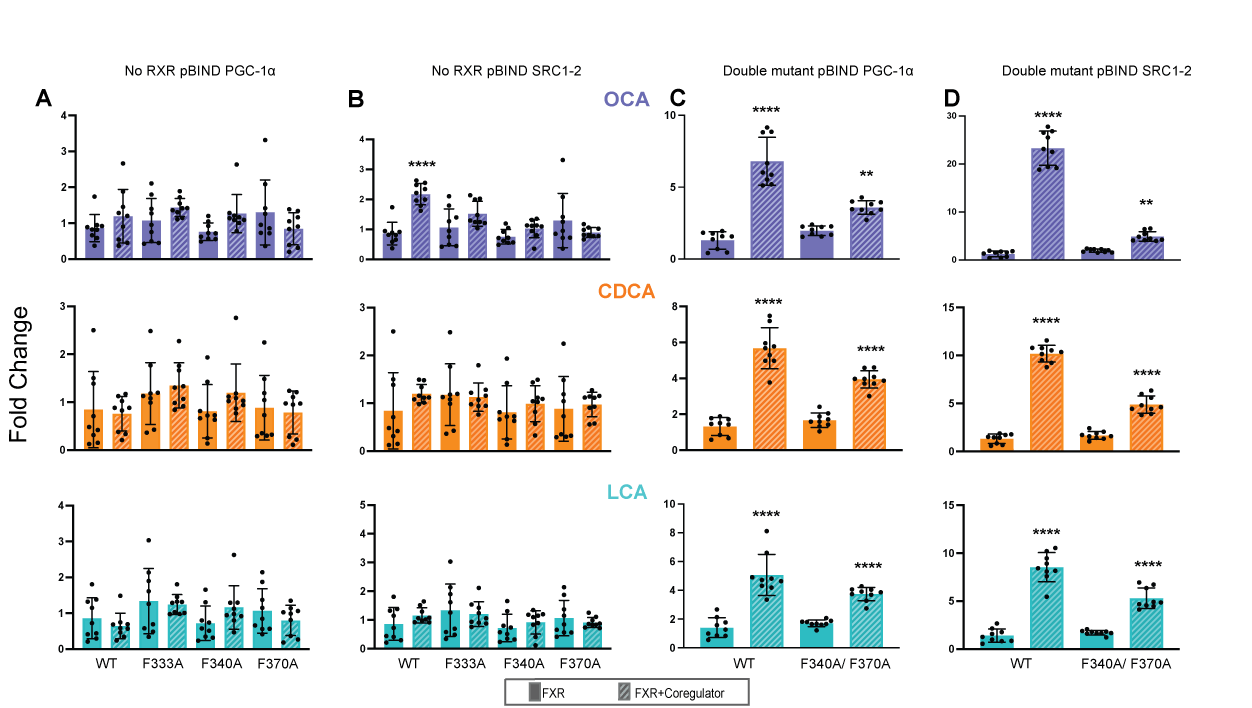


**Figure S4.** **The effect of H5 and H7 mutants on coregulator and RXR interaction with FXR using M2H assays.** **(A)** VP16-FXR-LBD luciferase with no added RXR. No interaction between PGC-1α and FXR was observed. **(B)** VP16-FXR-LBD luciferase with no added RXR. Only interaction between WT FXR-LBD and SRC1-2 was observed in OCA group. **(C)** H5+H7 double mutant luciferase, including PGC-1α and RXR. These mutations had no impact on FXR-LBD and PGC-1α interaction. **(D)** H5+H7 double mutant luciferase including SRC1-2 and RXR. These mutations had no impact on FXR-LBD and SRC1-2 interaction.


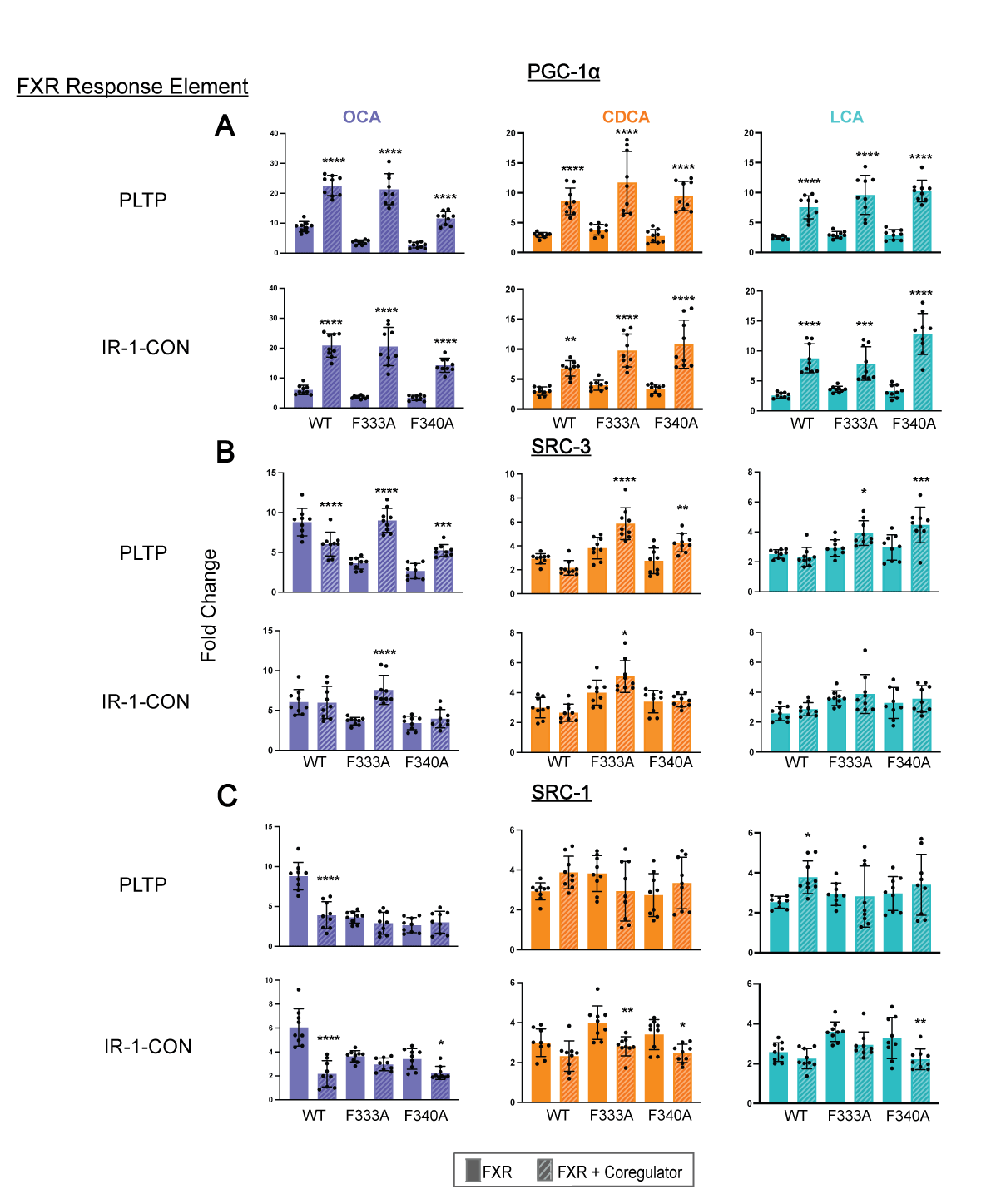


**Figure S5. Luciferase with FL-FXR and Coregulators.** **(A)** Luciferase with PGC-1α transfected into HeLa cells, no impact was seen on FXR interaction with PGC-1α with the H5 mutants. **(B)** Luciferase with SRC-3 transfected into HeLa cells. **(C)** Luciferase with SRC-1 transfected into HeLa cells.


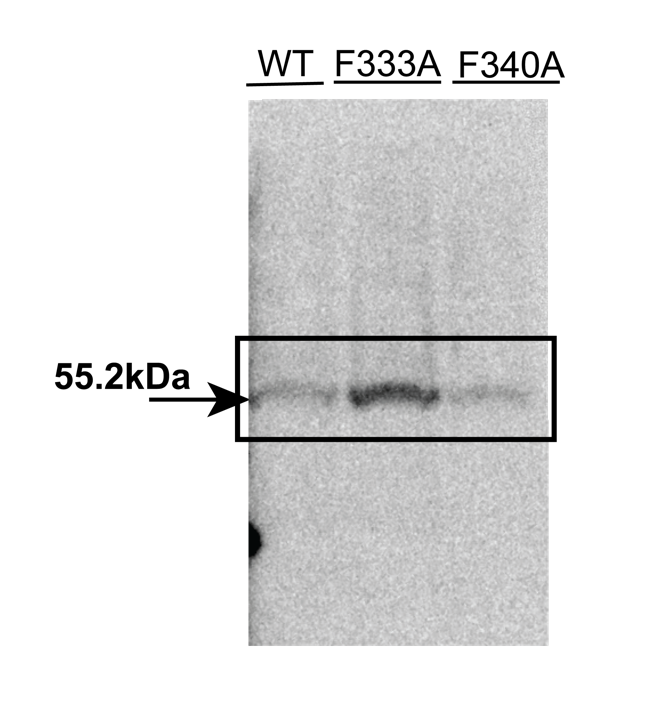


**Figure S6. Western blot analysis**. F340A mutation does not affect the expression of FXR in luciferase assays. Bands corresponding to the FXR protein at the expected molecular weight of 55.2 kDa are observed for WT, F333A, F340A.


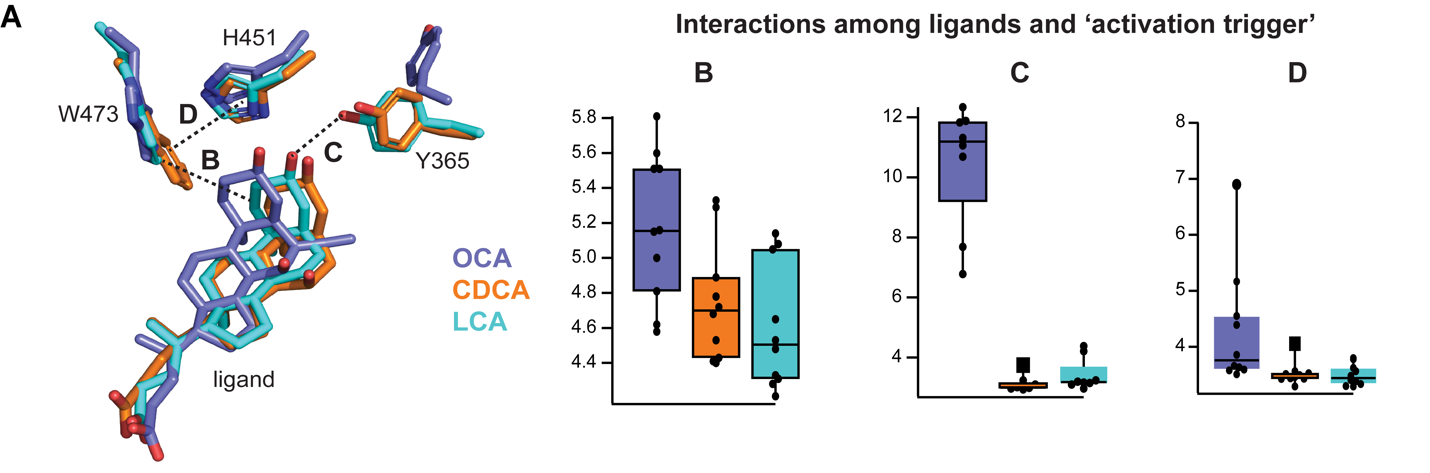


**Figure S7. Ligand interactions with ‘activation trigger’ residues in FXR**. The box plots (B), (C), and (D) representing the distances among ligands and the three residues (Y365, H451, W473) in different bile acid complexes. In all cases, OCA complexes display the highest distances.

**
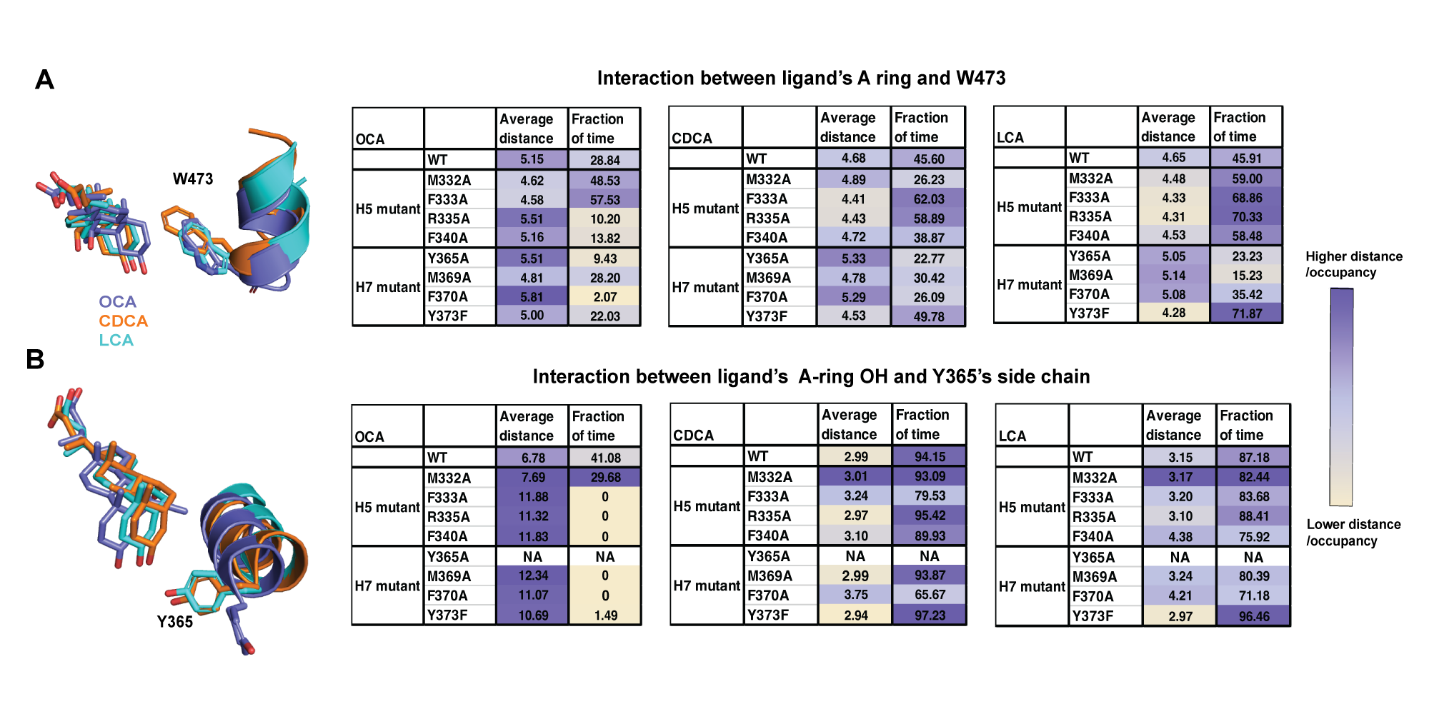
**

**Figure S8. Interactions between bile acids and other residues, W473 and Y365, in the binding pocket of FXR.** The average distance and the fraction of time of the interaction between WT, H5, and H7 mutants. **(A)** For OCA complexes, the interaction with W473 shows a larger average distance and lower occupancy compared to CDCA and LCA, indicating OCA binds to the receptor differently than the other bile acids. **(B)** The interaction between Y365 and the A-ring OH group of OCA complexes is significantly reduced, reflected by larger distances and lower occupancy compared to CDCA and LCA complexes. Color gradient represents the distance/occupancy of the interactions, with higher values in darker blue and lower values in lighter beige.


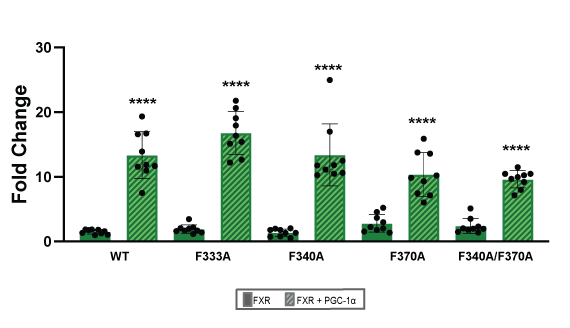


**Figure S9. Effect of H5 and H7 mutations on FXR and PGC-1α interaction in GW4064 treated cells.** The H5, H7, and double mutation had no impact on the interaction between FXR and PGC-1α with 10 µM GW4064.
